# Supplementary material for: Detecting stoichiometry of macromolecular complexes in live cells using FRET
Source: Nat Commun. 2016 Dec 6;7:13709. doi: 10.1038/ncomms13709 (PMC5150656; doi:10.1038/ncomms13709)
Supplement: Supplementary Information — Supplementary Figures 1-9, Supplementary Table 1, Supplementary Notes 1-3 and Supplementary References 1-5 [file ncomms13709-s1.pdf]

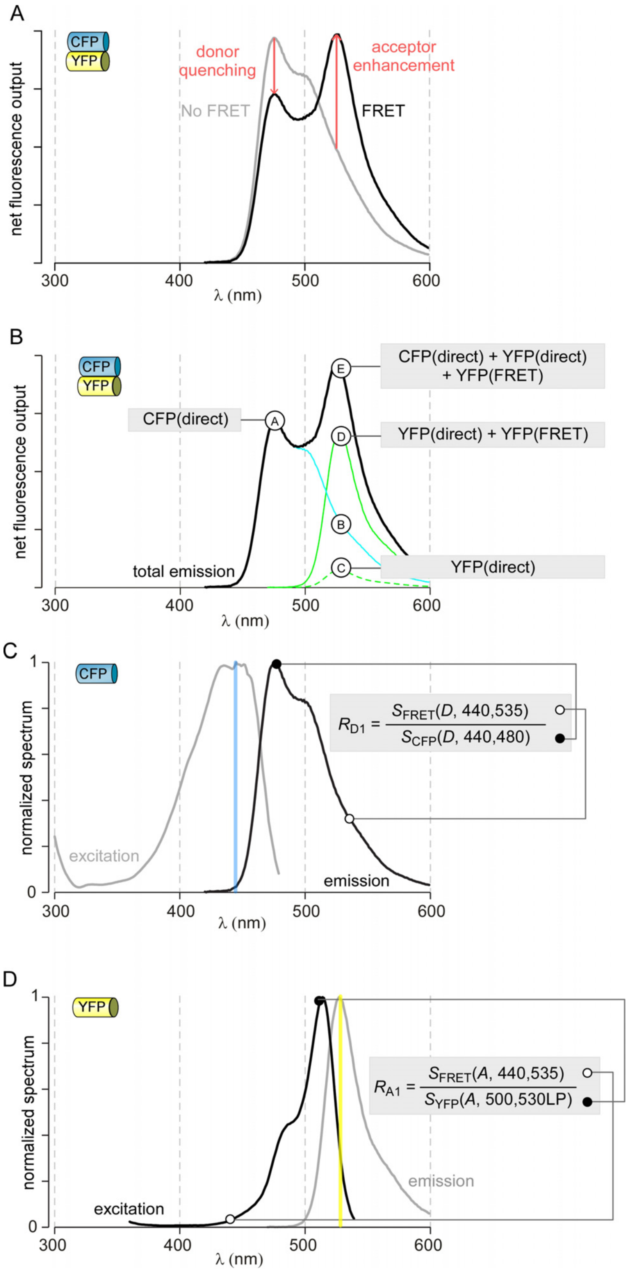

Supplementary Fig. 1| Unscrambling spectral cross-talk to measure FRET efficiency
a, Simulated total fluorescence emission spectrum of a 1:1 ECFP–EYFP complex in the presence (black) and absence (gray) of FRET ($E = 0.25$) when excited using 440 nm light. With FRET, the peak donor fluorescence is diminished while the peak acceptor fluorescence is enhanced. **b,** Sensitized emission can be estimated from the fluorescence output measured at 535 nm by subtracting various corrupting factors. The total fluorescence (point E) is the sum of emission from EYFP excited due to FRET, emission from EYFP excited by direct means (point C), and emission from ECFP molecules (point B). **c,** The ECFP emission can be measured with minimal corruption at 480 nm. The component of ECFP emission at 535 nm is deduced by defining the spectral factor R_{D1} as shown. Typically $R_{D1} \sim 0.33$. **d,** EYFP molecules can be excited specifically using 500 nm light. Accordingly, to estimate fluorescence emission at 535 nm from EYFP molecules that were excited directly, the spectral factor R_{A1} is defined as shown.

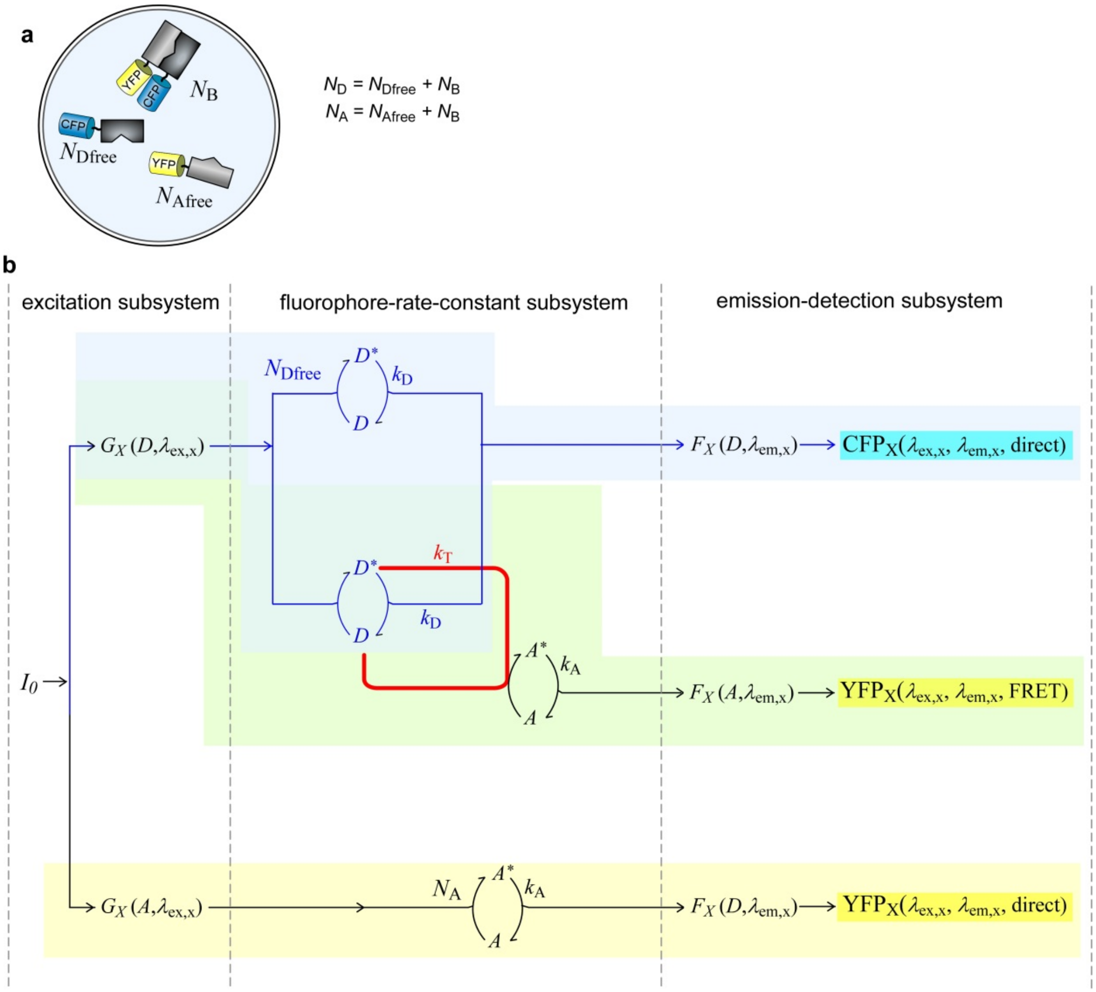

Supplementary Fig. 2 | Measurement of 3^3-FRET and E-FRET efficiencies for 1:1 binding interactions
a, Diagram conceptualizes a multimeric interaction. Here, N_{Dfree} corresponds to the concentration of unbound donors; N_{Afree} represents concentration of the unbound acceptors; N_{B} represents the total number of bound complexes. **b**, Complete state transition diagram specifies fluorescent output measurements necessary to determine both 3^3-FRET and E-FRET. The rate k_{T} specifies the rate of energy transfer between D and A molecule in each bound complex.

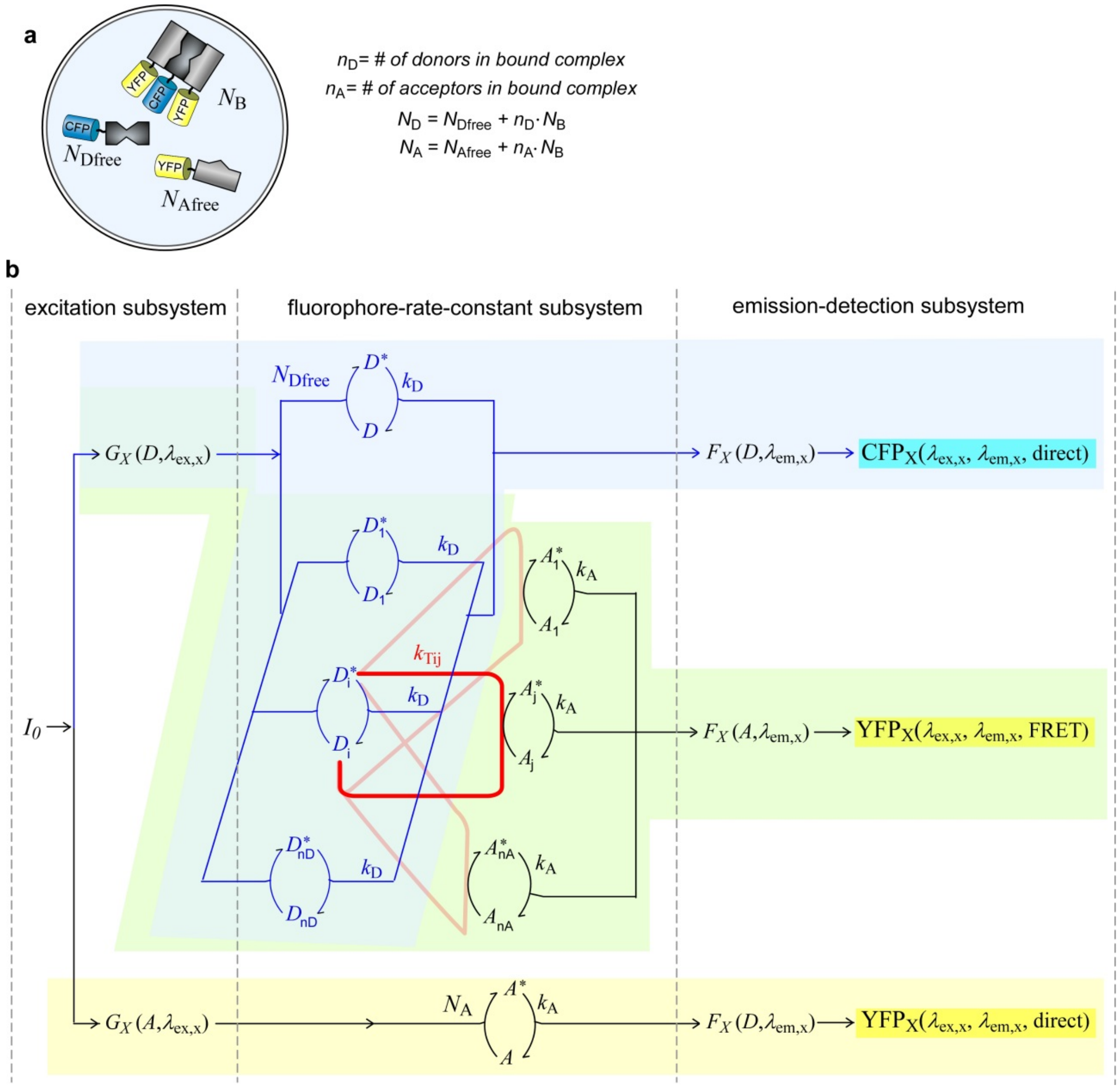

Supplementary Fig. 3 | Measurement of 3^3-FRET and E-FRET efficiencies for multimeric interactions
a, Diagram conceptualizes a multimeric interaction. Here, N_{Dfree} corresponds to the concentration of unbound donors; N_{Afree} represents concentration of the unbound acceptors; N_{B} represents the total number of bound complexes. **b**, Complete state transition diagram specifies fluorescent output measurements necessary to determine both 3^3-FRET and E-FRET. Notice here that n_{D} donor molecules are bound to n_{A} acceptor molecules and each donor-acceptor pair could undergo FRET with a particular rate of energy transfer k_{Tij}. For clarity, only the energy-transfer interactions emanating from the ith donor is depicted. Note that k_{Tij} could equal 0 for some fluorophore pairs depending upon their orientation and distance of separation.

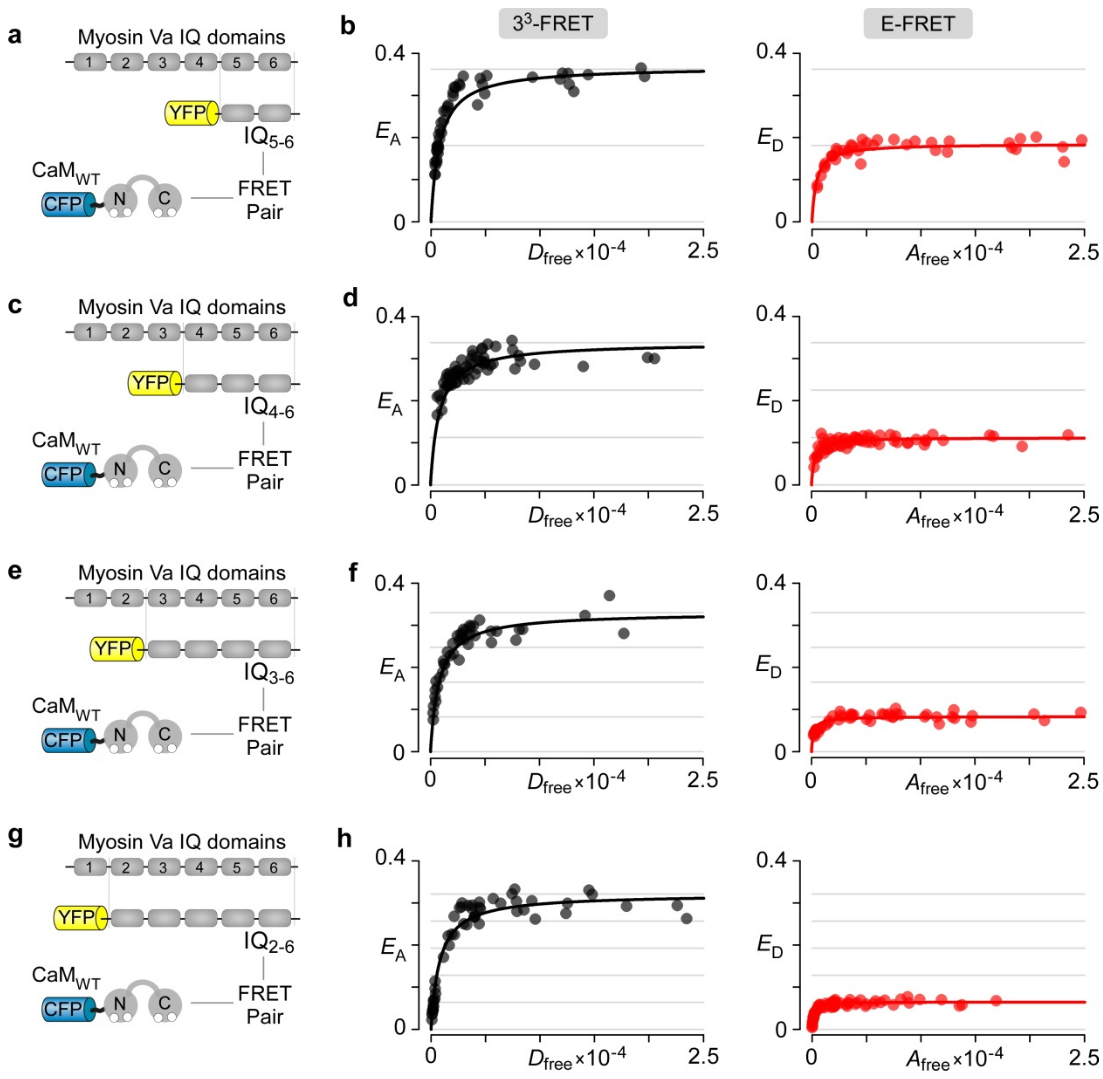

Supplementary Fig. 4 | Extended data for stoichiometry of CaM interaction with myosin Va neck domain. **a**, Schematic illustrates FRET binding pairs ECFP-fused CaM with EYFP-tagged myosin Va peptide containing two IQ domains ($IQ_{5\text{-}6}$). **b**, Left, 3^3-FRET efficiency (E_A) measured from cells co-expressing EYFP-$IQ_{5\text{-}6}$ with ECFP-CaM. Each black symbol corresponds to E_A deduced from a single cell plotted against the estimated free donor concentration (D_{free}). Right, E-FRET efficiencies (E_D) measured from the same cells are plotted as a function of estimated free acceptor concentrations (A_{free}). Here, the maximal 3^3-FRET efficiency ($E_{A,max}$) ~ 2× higher than the maximal E-FRET efficiency ($E_{A,max}$) suggesting that two CaM interact with this peptide. **c–d**, 3^3-FRET and E-FRET binding curves for ECFP-CaM binding to EYFP-tagged myosin Va peptide containing three tandem IQ domains. Format as in panels **a-b**. Note that the saturating 3^3-FRET efficiency is ~ 3× higher than the maximal E-FRET efficiency arguing that three CaM bind to this truncation. **e–f**, 3^3-FRET and E-FRET binding curves for ECFP-CaM binding to EYFP-fused myosin Va peptide containing four tandem IQ domains ($IQ_{3\text{-}6}$). Format as in panels **a-b**. The maximal 3^3-FRET efficiency is ~ 4× higher than the maximal E-FRET efficiency suggesting four CaM interact with this peptide. **g–h**, 3^3-FRET and E-FRET binding curves for ECFP-CaM binding to YFP-fused myosin Va peptide containing five tandem IQ motifs ($IQ_{2\text{-}6}$). Format as in panels **a-b**. In this case, the maximal 3^3-FRET efficiency is ~ 5× higher than the maximal E-FRET efficiency arguing that five CaM bind to this truncated peptide.

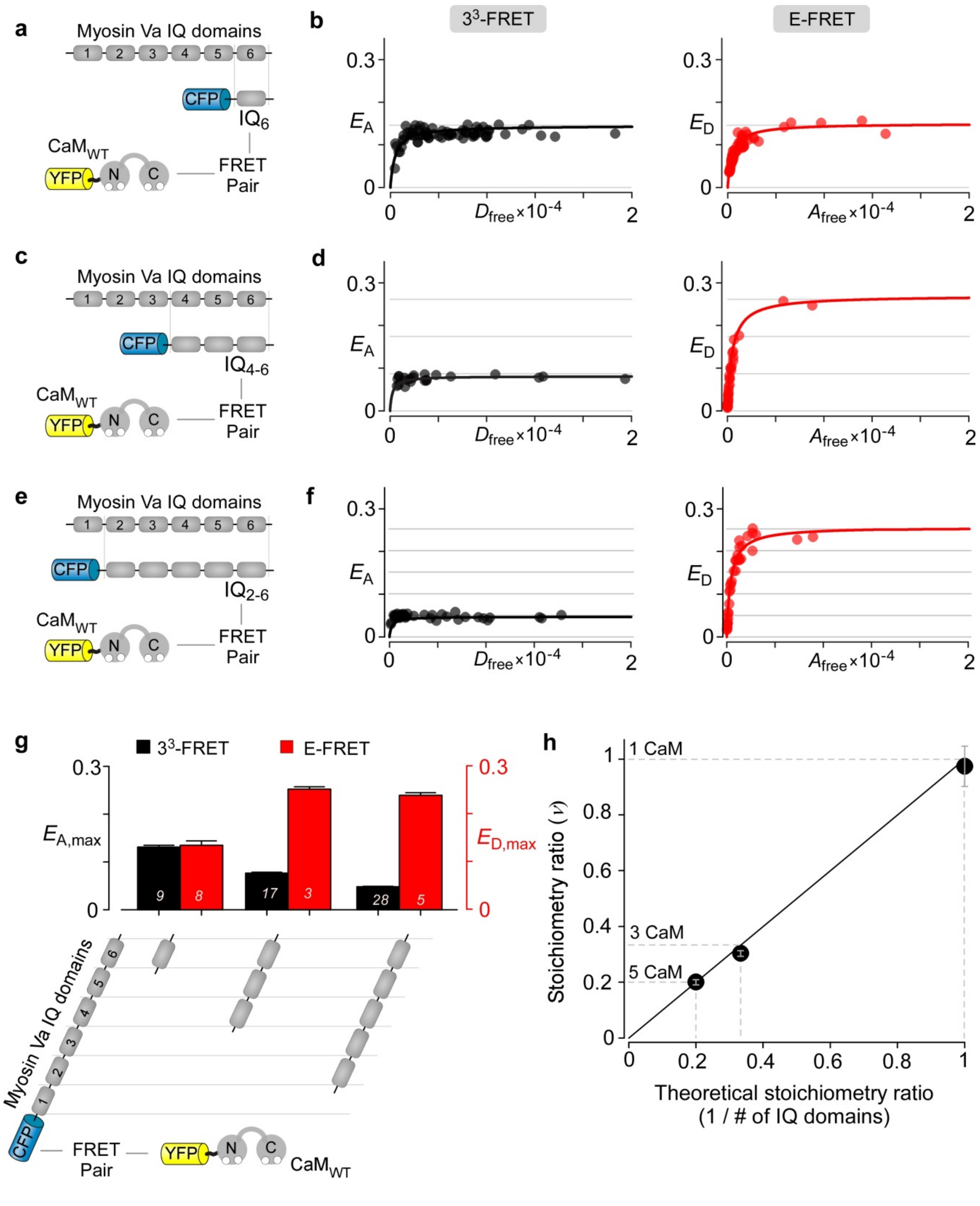

Supplementary Fig. 5 | Further validation of stoichiometry of CaM binding to myosin Va neck domain. **a**, Schematic illustrates FRET binding pairs EYFP-tagged CaM and ECFP tagged myosin Va peptide containing a single IQ domain (IQ_6). Note that the swapped fluorophore arrangement compared to main text Fig. 3, implies that the stoichiometry ratio, $\nu = n_D / n_A$ = 1/(# of CaM bound per myosin Va peptide) = 1/(# of IQ domains). **b**, Left, 3^3-FRET efficiency (E_A) is plotted against estimated free donor concentration (D_{free}). Each black symbol corresponds to data from a single cell. Right, E-FRET efficiency (E_D) is plotted as a function of estimated free acceptor concentration (A_{free}). As with main text Fig. 3**a-b**, the maximal 3^3-FRET efficiency ($E_{A,max}$) is approximately equal to maximal E-FRET efficiency ($E_{D,max}$) suggesting that a single CaM binds to a single IQ

peptide. **c–d**, 3^3-FRET and E-FRET binding data for for interaction of EYFP-fused CaM with ECFP fused myosin Va peptide containing three tandem IQ domains ($IQ_{4\text{-}6}$). Note that maximal 3^3-FRET efficiency is ~ 1/3 maximal E-FRET efficiency, consistent with three CaM (acceptor) bound to a single myosin Va $IQ_{4\text{-}6}$ peptide (donor). **e-f,** 3^3-FRET and E-FRET binding data for interaction of EYFP-fused CaM with ECFP fused myosin Va peptide containing five tandem IQ domains ($IQ_{2\text{-}6}$). Note that maximal 3^3-FRET efficiency is ~ 1/5 maximal E-FRET efficiency, consistent with five CaM (acceptor) bound to a single myosin Va $IQ_{4\text{-}6}$ peptide (donor). **g**, Bar-graph summarizes the maximal 3^3-FRET and E-FRET efficiencies for each of the FRET pairs shown in panels. Each bar corresponds to mean ± s.e.m with *n* as labeled. **a-f.** Note that the E-FRET efficiencies are larger than 3^3-FRET efficiencies for the multimeric interactions. This trend is a reversal of that shown in main text Fig. 3. **h**, Experimentally determined stoichiometry ratio, $\nu = E_{A,max}/E_{D,max}$ follows the identity relationship with the expected ratio of donor to acceptors in each myosin Va truncation. Note that since CaM is tagged with an acceptor fluorophore, the theoretical stoichiometry ratio $\nu = n_D/n_A$ = 1/(# of CaM) = 1 / (# of IQ motifs).

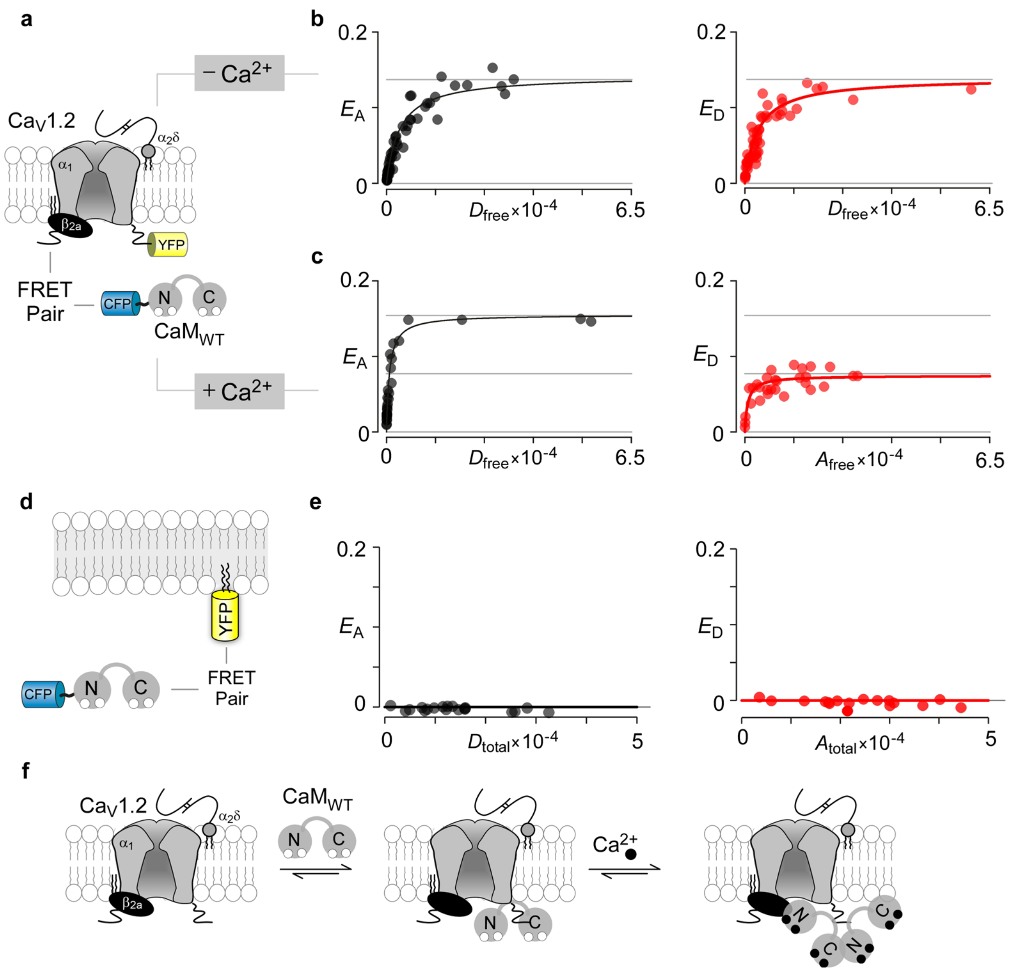

Supplementary Fig. 6 | Extended data for stoichiometry of CaM interaction with Ca_V1.2 channels

a, Schematic illustrates FRET binding pairs ECFP tagged CaM and Ca_V1.2 holochannel fused with EYFP on its carboxy-terminus. For these experiments, the Ca^{2+} channel auxiliary subunits β_{2A} and $\alpha_2\delta$ are coexpressed to reflect native Ca^{2+} channel complexes. **b**, Under resting cytosolic Ca^{2+} levels, apoCaM (Ca^{2+}-free CaM) interacts with the Ca_V1.2 with a 1:1 stoichiometry. Left, 3^3-FRET efficiency (E_A) is plotted against estimated free donor concentration (D_{free}). Each black symbol corresponds to data from a single cell. Right, E-FRET efficiency (E_D) is plotted as a function of estimated free acceptor concentration (A_{free}). As evident, the maximal 3^3-FRET efficiency ($E_{A,max}$) is approximately equal to maximal E-FRET efficiency ($E_{D,max}$). **c**, Upon increasing the cytosolic Ca^{2+} concentration by application of ionomycin, the stoichiometry of CaM interaction with L-type channel complex doubles. Format as in panel **b**. Note that maximal 3^3-FRET efficiency is ~ 2× higher than maximal E-FRET efficiency arguing for the presence of two donor molecules (CaM) for each acceptor molecule (channel) in the bound complex. **d**, Cartoon illustrates negative control FRET binding pairs ECFP tagged CaM and EYFP tethered to the membrane via a farnesylation. **e,** No appreciable FRET is detected between ECFP tagged CaM and EYFP-tethered to membrane. Left, 3^3-FRETefficiency (E_A) is plotted against total donor concentration. Right, E-FRET efficiency (E_D) is plotted against total acceptor concentration. **f**, Cartoon illustrates scheme of CaM binding to the L-type Ca^{2+} channel complex. At low cytosolic Ca^{2+} levels, a single apoCaM preassociates to the channel complex. Upon Ca^{2+} elevation, a second CaM is recruited to the channel complex.

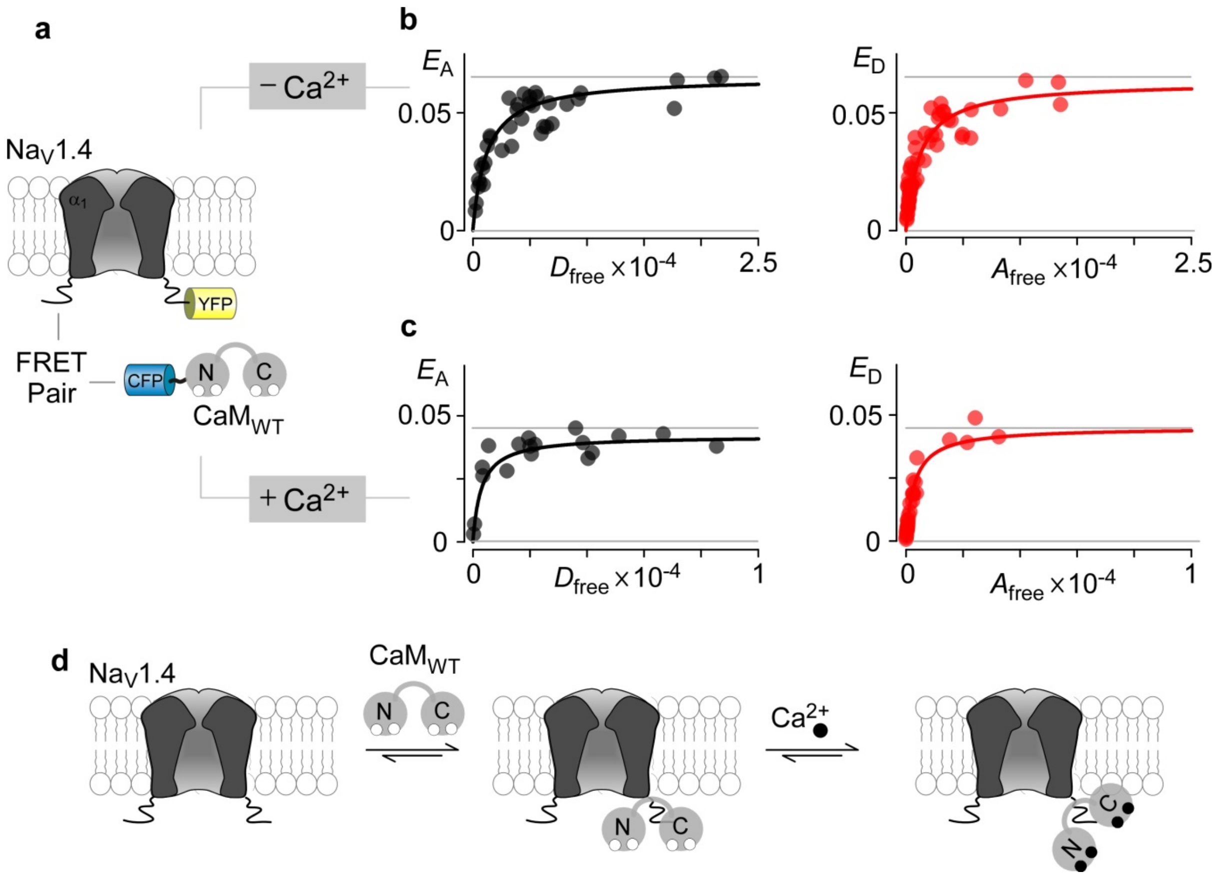

Supplementary Fig. 7 | Extended data for stoichiometry of CaM interaction with Na_V1.4 channels.
a, Cartoon shows FRET binding pairs ECFP tagged CaM and Na_V1.4 holochannel fused with EYFP on its carboxy-terminus. **b**, Under resting cytosolic Ca^{2+} levels, the saturating 3^3-FRET efficiency is ~ saturating E-FRET efficiency arguing that a single apoCaM binds to Na_V1.4 channel complex. Format as in Supplementary Fig. 3**a-b**. **c**, For the Na_V1.4 channels, Ca^{2+}/CaM also associates with a 1:1 stoichiometry. As evident, even under elevated cytosolic Ca^{2+} concentrations, the maximal 3^3-FRET and E-FRET efficiencies remain approximately equal to each other. **d**, Schematic summarizes the scheme of CaM interaction with Na_V1.4 channels. For these Na channels, a single apoCaM preassociates to the channel complex, and a single CaM remains bound to the channel complex even under elevated Ca^{2+} conditions.

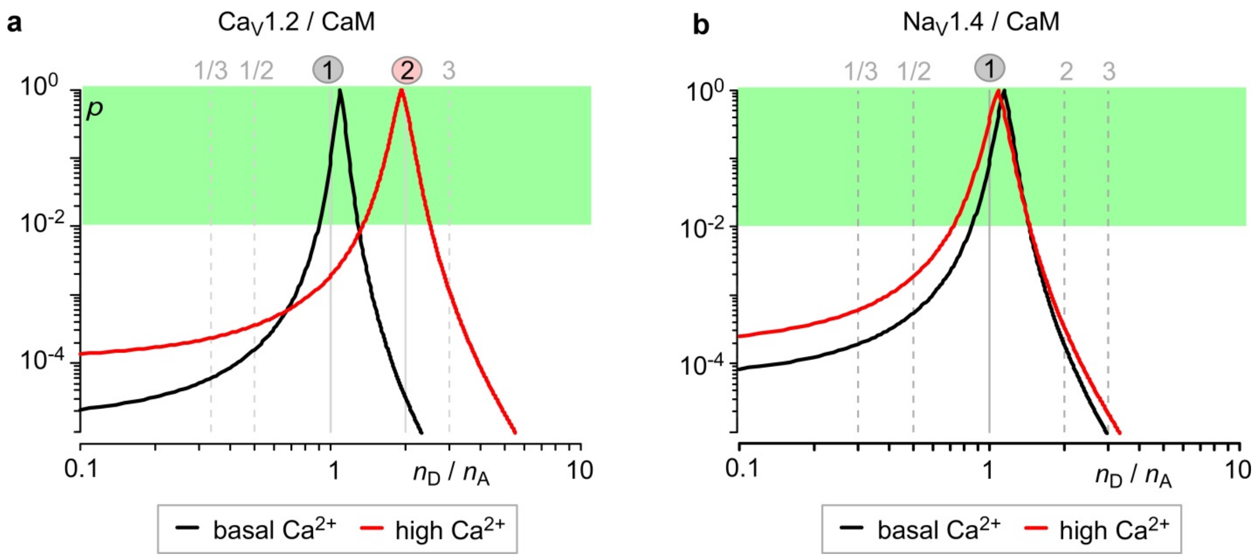

Supplementary Fig. 8 | Extended statistical analysis shows Ca^{2+}-induced switching of CaM stoichiometry with $Ca_V1.2$ but not $Na_V1.4$.

a, Graph displays the probability that the experimentally observed $E_{A,max}$ and $E_{D,max}$ values for the interaction of CFP-tagged CaM with YFP-tagged $Ca_V1.2$ holochannel correspond to a given binding stoichiometry. Under basal Ca^{2+} conditions (black curve), the likelihood that the data represents to a 1:1 CaM/channel interaction is 100-1000× more likely than a 2:1 or 1:2 stoichiometry. Under elevated cytosolic Ca^{2+} conditions (red curve), the data are consistent with a 2:1 stoichiometry with a 1000 likelihood than a 1:1 stoichiometry indicating a Ca^{2+}-dependent shift in stoichiometry. **b,** For interaction of CFP-tagged CaM with YFP-tagged $Na_V1.4$ channel under both basal and elevated Ca^{2+} conditions, $E_{A,max}$ and $E_{D,max}$, values were consistent with a 1:1 stoichiometry with a 1000× likelihood over a potential 2:1 stoichiometry. This outcome substantiates the absence of a Ca^{2+}-induced shift in CaM stoichiometry for the Na channel.

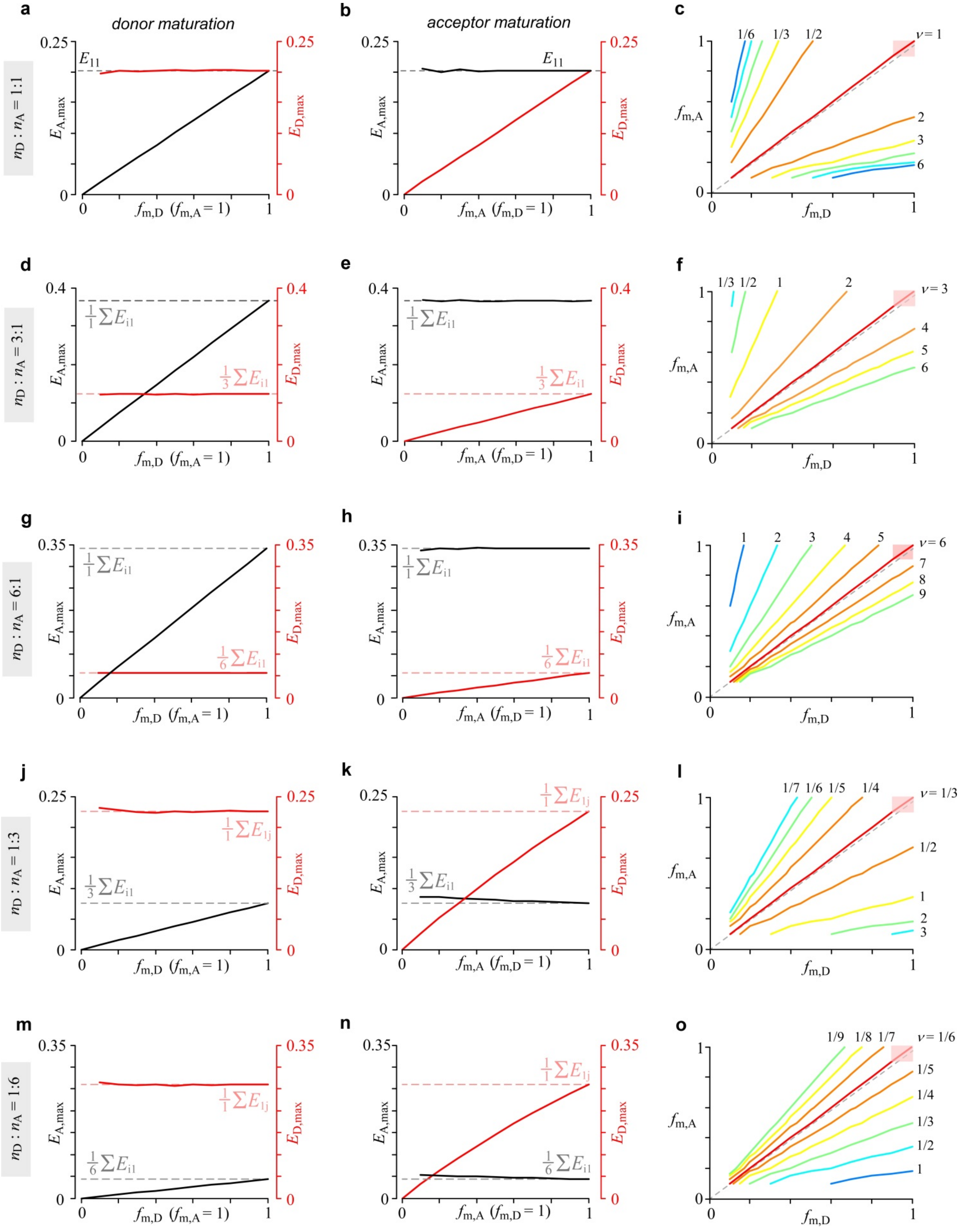
a
donor maturation
b
acceptor maturation
c
$n_D : n_A = 1:1$
$n_D : n_A = 3:1$
$n_D : n_A = 6:1$
$n_D : n_A = 1:3$
$n_D : n_A = 1:6$
d
e
f
g
h
i
j
k
l
m
n
o
$E_{A,max}$
$E_{D,max}$
$f_{m,D}$ ($f_{m,A}$ = 1)
$f_{m,A}$ ($f_{m,D}$ = 1)
$f_{m,A}$
$f_{m,D}$
E_{11}
$\frac{1}{1}\sum E_{i1}$
$\frac{1}{3}\sum E_{i1}$
$\frac{1}{6}\sum E_{i1}$
$\frac{1}{1}\sum E_{1j}$
$\nu = 1$
$\nu = 3$
$\nu = 6$
$\nu = 1/3$
$\nu = 1/6$

Supplementary Fig. 9 | Effect of immature donor and acceptor fluorophores on $E_{A,max}$ and $E_{D,max}$

a, Stochastic simulation shows the effect of immature donors on maximal 3^3-FRET and E-FRET efficiencies for 1:1 complexes. Black, $E_{A,max}$ is linearly proportional to the fraction of mature donors ($f_{m,D}$). Red, $E_{D,max}$ is unperturbed by the presence of immature donors. **b**, The effect of immature acceptors on maximal $E_{A,max}$ and $E_{D,max}$. Incomplete folding of the acceptors results in a proportionate decrease in $E_{D,max}$. By contrast, $E_{A,max}$ is insensitive to acceptor maturation. **c**, Contour plot shows stoichiometry ratios (ν) as a function of donor and acceptor maturation for 1:1 complexes. If the maturation efficiency of the donors and acceptors are similar ($f_{m,D} \sim f_{m,A}$), then the ratio $E_{A,max}$ / $E_{D,max}$ is a reliable estimator for interaction stoichiometry (red contour line). The relative maturation rate for ECFP-EYFP pair is plotted as gray dashed line. Red shaded area corresponds to region where both donor and acceptor have greater than 90% folding efficiency. In this range, the assay would accurately report 1:1 interaction stoichiometry. **d-e**, Simulation shows $E_{A,max}$ and $E_{D,max}$ for 3:1 complexes in the presence of immature of donors and immature acceptors. Format as in panel **a-c**. $E_{A,max}$ is linearly dependent on $f_{m,D}$, and $E_{D,max}$ is linearly proportional to $f_{m,A}$. **f**, Contour plot shows that the stoichiometry ratio (ν) correctly identifies interaction stoichiometry if $f_{m,A}$, $f_{m,D}$ > 0.9 (red shaded area). **g-i,** Simulation shows effect of incomplete maturation of donors and acceptors on $E_{A,max}$ and $E_{D,max}$ for 6:1 complexes. Format as in panels **a-c**. The assay can resolve a 6:1 stoichiometry if the maturation efficiencies $f_{m,A}$, $f_{m,D}$ > 0.9. **j-l**, The effect of incomplete maturation of donors and acceptors on $E_{A,max}$ and $E_{D,max}$ for 1:3 complexes. Format as in panels **a-c**. As observed for complexes with multiple donors, $E_{D,max}$ is largely insensitive to $f_{m,D}$ while highly-dependent on $f_{m,A}$. By contrast $E_{A,max}$ is linearly dependent on $f_{m,D}$. Reassuringly, in the range that $f_{m,A}$, $f_{m,D}$ > 0.9 (red shaded area), the stoichiometry ratio is a reliable reporter of interacting stoichiometry. **m-o.** $E_{A,max}$ and $E_{D,max}$ for 1:6 complexes shows similar trend with partial maturation of fluorophores. For $f_{m,A}$, $f_{m,D}$ > 0.9, ratio of maximal FRET efficiencies is a reliable reporter of interaction stoichiometry.

	Binding Pair (CFP-CaM + YFP-x)	Live-cell relative affinity $K_{d,EFF}$ (D_{free}) [a]
**1**	YFP-IQ_6 (**Fig. 3b**)	800 D_{free}
**2**	YFP-IQ_{5-6} **(Fig. S4b)**	800 D_{free}
**3**	YFP-IQ_{4-6} **(Fig. S4d)**	800 D_{free}
**4**	YFP-IQ_{3-6} **(Fig. S4f)**	800 D_{free}
**5**	YFP-IQ_{2-6} **(Fig. S4h)**	800 D_{free}
**6**	YFP-IQ_{1-6} **(Fig. 3d)**	800 D_{free}
**7**	Ca_V1.2-YFP (- Ca) **(Fig. S6b)**	3500 D_{free}
**8**	Ca_V1.2-YFP (+ Ca) **(Fig. S6c)**	700 D_{free}
**9**	Na_V1.4-YFP (- Ca) **(Fig. S7b)**	1200 D_{free}
**10**	Na_V1.4-YFP (+ Ca) **(Fig. S7c)**	150 D_{free}

Supplementary Table 1| Relative dissociation constants recovered from FRET 2-hybrid binding assay.
[a]$K_{d,EFF}$ reports relative dissociation constants determined from FRET 2-hybrid assay. The relative dissociation constants can be converted to estimated K_d in nanomolars by the relation $K_{d,EST} = 0.0326 \times K_{d,EFF}$ based on ref. 3.

SUPPLEMENTARY NOTE 1

Derivation of stoichiometry ratio based on FRET efficiencies from multimeric complexes

Upon excitation by light, a fluorophore typically relaxes by emitting a photon with a certain probability or quantum efficiency. However, this fluorophore could also de-excite by non-radiative energy transfer to a nearby fluorophore. In this context, the fluorophore that transferred energy is referred to as the 'donor' and the fluorophore that received energy is referred to as the 'acceptor.' This process called Förster Resonance Energy Transfer or Fluorescence Resonance Energy Transfer (FRET) depends crucially upon both spatial arrangement of the two fluorophores and also the degree of overlap between the emission spectrum of the donor and absorption spectrum of the acceptor. As the emission spectrum of the acceptor is redshifted compared to the donor, the efficacy of FRET could be assessed by characterizing the spectrum of the donor-acceptor complex. **Supplementary Fig. 1a** simulates the effect of FRET on the total fluorescence emission spectrum for an ECFP-EYFP dimer excited by 440 nm light. In the presence of FRET (efficiency, E = 0.25), the peak of the total emission spectrum at λ = 480 nm (~ peak of donor or ECFP emission) is quenched. Instead, a new peak is now prominent at λ = 535 nm (~ peak emission of acceptor or EYFP). These spectral changes lend naturally to two metrics of FRET efficiencies: (1) donor-centric FRET efficiency (E_D) characterizes the fractional quenching of the donor fluorescence. (2) Acceptor-centric FRET efficiency (E_A) quantifies the acceptor emission induced as a result of FRET from the donor – termed sensitized emission, normalized to the total emission if the acceptor was excited through direct means. The asymmetry in these metrics can be used to determine the ratio of donors to acceptors in a given complex.

Practically, the characterization of the FRET-associated spectral changes is confounded by the broad overlapping excitation and emission spectra of the donor and acceptor fluorescent proteins (**Supplementary Fig. 1b**). Recent studies have, however, devised robust live-cell strategies to deduce both donor- and acceptor-centric FRET efficiencies non-destructively using fluorescence measurements obtained using three distinct filter cubes. As outlined below for ECFP/EYFP FRET pairs, the 3^3-FRET method[1] deduces acceptor-centric FRET efficiencies using fluorescence measurements through CFP, YFP, and FRET cubes. Similarly, the E-FRET method[2] quantifies donor-centric FRET efficiencies using the same three fluorescence measurements.

1.1 Experimental Determination of 3^3-FRET and E-FRET Efficiencies.

1.1.1 *Filter cube specifications and typical fluorescence measurements.*

To determine FRET efficiencies by 3^3-FRET method or E-FRET method, fluorescence measurements are obtained from isolated single cells expressing both donor (ECFP-tagged molecule) and acceptor (EYFP-tagged molecule) pair using three filter cubes:

(1) The CFP cube has an excitation filter centered near the peak of ECFP absorption spectrum (λ_{ex} ~440 nm) and emission filter centered near peak ECFP emission (λ_{em} ~480 nm). For our setup, we utilize excitation filter D440/20M (Chroma), dichroic mirror 455DCLP (Chroma), and emission filter D480/30M (Chroma).

(2) The YFP cube has its excitation filter centered near the peak of EYFP absorption spectrum (λ_{ex} ~ 500 nm) and emission filter well suited to capture EYFP emission (λ_{em} ~ 535 nm). For our setup, we use excitation filter 500RDF25 (Omega Optical), dichroic mirror 525DRLP (Omega Optical), and emission filter 530EFLP (Omega Optical).

(3) The FRET cube, like the CFP cube, has its excitation near the peak of ECFP absorption spectrum (λ_{ex} ~ 440 nm) but its emission is chosen to capture EYFP emission (λ_{em} ~ 535 nm). For our setup, we use excitation filter 440DF20 (Omega Optical), dichroic mirror 455DRLP (Omega Optical), and emission filter 535DF25 (Omega Optical).

In addition, for the purposes of determining spectral ratios to subtract various corrupting factors that confound FRET efficiency computation, fluorescence measurements are also obtained from cells expressing ECFP alone (R_{D1} and R_{D2} defined in **Supplementary Fig. 1c**) and EYFP alone (R_{A1} defined in **Supplementary Fig. 1d**).

1.1.2 *Notation for fluorescence measurements and other constants*

For concreteness, we here follow the notation presented in Erickson et al[1]:

(1) $XFP_x(\lambda_{ex,x}, \lambda_{em,x}$, mode of excitation) – the fluorescence output for a given fluorophore ($XFP \in$ {CFP; YFP}) as measured with a particular cube 'x'. In general, the mode of excitation is either 'direct' or 'FRET.' For instance, the fluorescence output of EYFP molecules that were stimulated through direct excitation when measured using FRET cube is denoted by $YFP_{FRET}(\lambda_{ex,FRET}, \lambda_{em,FRET}$, direct). Likewise, the fluorescence output of EYFP excited as a result of FRET from ECFP when measured using FRET cube is represented by $YFP_{FRET}(\lambda_{ex,FRET}, \lambda_{em,FRET}$, FRET) or YFP_{FRET}(440, 535, FRET).

(2) $S_x(specimen, \lambda_{ex,x}, \lambda_{em,x})$ – the actual fluorescence signal output obtained from a given sample using a certain optical cube 'x'. Here *specimen* refers to the composition of fluorophores in the given cell. In general, *specimen* $\in$ {D, donor alone; A, acceptor alone; DA, both donors and acceptors}. For example, the signal output of cells containing ECFP alone measured through FRET cube is given by $S_{FRET}(D, \lambda_{ex,FRET}, \lambda_{em,FRET})$ or S_{FRET}(D, 440, 535).

Constants specifying excitation and emission of donor and acceptor fluorophores

(3) $G_x(y, \lambda_{ex,x})$ – this constant specifies the excitation properties of the fluorophore $y \in$ {D, donor; A, acceptor}) when excitation was attained using cube 'x'. This constant incorporates spectral properties of the lamp, optical properties of the excitation filter and dichroic mirror of filter cube x, and wavelength-dependent absorption properties of the fluorophore y as given by a molar extinction coefficient, $\varepsilon_y(\lambda)$. For example, $G_{FRET}(D, \lambda_{ex,FRET})$ or G_{FRET}(D, 440) would represent the excitation properties of ECFP measured using the FRET cube.

(4) $F_x(y, \lambda_{em,x})$ – this constant specifies the fluorescence output of fluorophore $y \in$ {D, donor; A, acceptor}) when measured using optical cube 'x'. This quantity is an "output transfer function" that converts donor or acceptor relaxations to mV experimentally recording using the PMT. This constant incorporates the emission spectrum and the quantum yield of the fluorophore *y*, the dichroic mirror and emission filter optical properties of cube 'x', and frequency-dependent sensitivity of the PMT detector. For convenience, we define $F_x(y, \lambda_{em,x}) = QY_y \cdot \hat{F}_x(y, \lambda_{em,x})$ to specify the quantum yield of fluorophore *y*. As an example, $F_{FRET}(A, \lambda_{em,FRET})$ or F_{FRET}(A, 535) represents the output transfer function for EYFP measured using FRET cube.

Spectral Ratios to isolate donor and acceptor signals in mixed specimen.

(5) R_{A1} – This constant is determined from cells expressing EYFP alone and allows for the conversion of "optically isolated" YFP signal, S_{YFP}(DA, 500, 530LP), to the contribution of directly-excited YFP to fluorescence measured using 440 nm light that excites both ECFP and EYFP molecules. The ratio is defined as:

$$R_{A1} = \frac{S_{FRET}(A, 440, 535)}{S_{YFP}(A, 500, 530LP)} = \frac{G_{FRET}(A, 440) \cdot F_{FRET}(A, 535)}{G_{YFP}(A, 500) \cdot F_{YFP}(A, 530LP)}$$

(6) R_{D1} – This constant is determined from cells expressing ECFP alone and allows for the conversion of "optically isolated" CFP signal, S_{CFP}(DA, 440, 480), to the contribution of CFP to fluorescence at 535 nm where both CFP and YFP are appreciable. The ratio is defined as:

$$R_{D1} = \frac{S_{FRET}(D, 440, 535)}{S_{CFP}(D, 440, 480)} = \frac{G_{FRET}(D, 440) \cdot F_{FRET}(D, 535)}{G_{CFP}(D, 440) \cdot F_{CFP}(D, 480)}$$

(7) R_{D2} – This constant is determined from cells expressing ECFP alone and allows for the conversion of "optically isolated" CFP signal, S_{CFP}(DA, 440, 480), to the contribution of CFP to fluorescence measured using YFP cube. The ratio is defined as:

$$R_{D2} = \frac{S_{YFP}(D, 500, 530LP)}{S_{CFP}(D, 440, 480)} = \frac{G_{FRET}(D, 500) \cdot F_{FRET}(D, 530LP)}{G_{CFP}(D, 440) \cdot F_{CFP}(D, 480)}$$

1.1.3 *Computation of 3^3-FRET efficiencies*

The 3^3-FRET method measures sensitized emission or the fractional increase in acceptor intensity due to FRET. As described at length by Erickson *et al*[1], the 3^3-FRET efficiency (E_A) is defined as,

$$E_A = \frac{YFP_{FRET}(\lambda_{ex,FRET}, \lambda_{em,FRET}, \text{FRET})}{YFP_{FRET}(\lambda_{ex,FRET}, \lambda_{em,FRET}, \text{direct})} \cdot \frac{G_{FRET}(A, \lambda_{ex,FRET})}{G_{FRET}(D, \lambda_{ex,FRET})} = \frac{YFP_{FRET}(440,535,\text{FRET})}{YFP_{FRET}(440,535,\text{direct})} \cdot \frac{G_{FRET}(A,440)}{G_{FRET}(D,440)} \quad \text{(S1.1)}$$

The factor $G_{FRET}(\text{A}, \lambda_{ex,FRET})/G_{FRET}(\text{D}, \lambda_{ex,FRET})$ is an instrument specific parameter that normalizes for the differences in the extinction coefficients of EYFP and ECFP at the wavelength $\lambda_{ex,FRET}$. For our experimental setup, $G_{FRET}(\text{A}, \lambda_{ex,FRET})/G_{FRET}(\text{D}, \lambda_{ex,FRET}) = 0.058$.

Experimentally, 3^3-FRET efficiency can be computed from fluorescence measurements from CFP ($S_{CFP}(DA, 440, 480)$), YFP($S_{YFP}(DA, 500, 530\text{LP})$), and FRET ($S_{FRET}(DA, 440, 535)$) filter cube measurements by the following formula:

$$E_A = \frac{S_{FRET}(DA,440,535) - R_{D1} \cdot S_{CFP}(DA,440,480) - R_{A1} \cdot S_{YFP}(DA,500,530LP)}{R_{A1} \cdot S_{YFP}(DA,500,530\text{LP})} \cdot \frac{G_{FRET}(A,440)}{G_{FRET}(D,440)} \quad \text{(S1.2)}$$

A comprehensive derivation and description of the 3^3-FRET formula presented in Eq. S1.2 can be found in Supplemental Material for Erickson *et al*[1]. Here, we provide a brief conceptual description for this relation.

In the ideal case, fluorescence measurement using the FRET cube, $S_{FRET}(DA, 440, 535)$, would yield $YFP_{FRET}(440, 535, \text{FRET})$ – the emission of EYFP as a result of FRET following ECFP excitation. However, since the excitation and emission spectra of ECFP and EYFP are broad and overlapping (**Supplementary Fig. S1b)**, this fluorescence measurement is instead composed of three components:

(1) fluorescence emission from EYFP excited by FRET from ECFP (the desired component)

(2) EYFP emission as a result of direct excitation (point *C*)

(3) ECFP emission as a result of spectral crosstalk (point *B*).

Thus,

$$S_{FRET}(DA,440,535) = YFP_{FRET}(440,535,\text{FRET}) + YFP_{FRET}(440,535,\text{direct}) + CFP_{FRET}(440,535,\text{direct}) \quad \text{(S1.3)}$$

The term $CFP_{FRET}(440, 535, \text{direct})$ corresponds to spectral crosstalk from ECFP molecules as a result of its broad emission spectrum. Fluorescence from ECFP can be determined using the CFP cube since EYFP emission at 480 nm light is negligible. Thus, we can estimate this component using the spectral correction factor R_{D1} and the "optically isolated," $S_{CFP}(\text{DA}, 440, 480)$ signal (**Supplementary Fig. S1c**).

$$CFP_{FRET}(440,535,\text{direct}) = R_{D1} \cdot S_{CFP}(\text{DA},440,480) \quad \text{(S1.4)}$$

The term $YFP_{FRET}(440, 535, \text{direct})$ is the fluorescence emission from EYFP molecules directly excited by the 440 nm light. This component is estimated from fluorescence measurements using YFP cube (S_{YFP}) and a spectral ratio R_{A1} (**Supplementary Fig. S1d**). Importantly, a very small fraction of ECFP molecules are also excited by the YFP cube. This fraction can be subtracted using the factor $R_{D2} = S_{YFP}(\text{D}, 505, 530\text{LP}) / S_{CFP}(\text{D}, 440, 480)$. Thus,

$$YFP_{FRET}(440,535,\text{direct}) = R_{A1} \cdot (S_{YFP}(\text{DA},500,530\text{LP}) - R_{D2} \cdot S_{CFP}(\text{DA},440,480)) \quad \text{(S1.5)}$$

Experimentally, $R_{D2} \sim 0.007$ and since cells chosen for FRET analysis are such that, $S_{CFP}(\text{DA},440,480) / S_{YFP}(\text{DA},500,530\text{LP}) < 10$,

$$YFP_{FRET}(440,535,\text{direct}) \approx R_{A1} \cdot S_{YFP}(500,530\text{LP}) \quad \text{(S1.6)}$$

Subtracting these corrupting factors, Eqs. S1.4 and S1.6, from FRET cube measurement, we obtain

$$YFP_{\text{FRET}}(440,535,\text{FRET}) = S_{\text{FRET}}(440,535) - R_{\text{D1}} \cdot S_{\text{CFP}}(440,480) - R_{\text{A1}} \cdot S_{\text{YFP}}(500,530\text{LP}) \quad \text{(S1.7)}$$

Eq. S1.2 can be derived by substituting Eqs. S1.6 and S1.7 into Eq. S1.1.

1.1.4 *Experimental computation of E-FRET efficiencies*

Donor-centric FRET efficiency (E_D) is defined as the fractional quenching of ECFP fluorescence due to FRET. Thus,

$$E_{\text{D}} = \frac{CFP_{\text{FRET|after}} - CFP_{\text{FRET|before}}}{CFP_{\text{FRET|after}}} \quad \text{(S1.8)}$$

where $CFP_{\text{FRET|before}}$ is the quenched ECFP fluorescence intensity in the presence of FRET, while $CFP_{\text{FRET|after}}$ refers to the ECFP fluorescence intensity in absence of FRET is typically measured as the intensity after all acceptors are photobleached.

The E-FRET method non-destructively estimates donor-centric FRET from live cells using fluorescence measurements using three filter cubes – CFP, YFP, and FRET cubes. The E-FRET efficiency is computed as,

$$E_{\text{D}} = \frac{YFP_{\text{FRET}}(\lambda_{\text{ex,FRET}}, \lambda_{\text{em,FRET}}, FRET)}{YFP_{\text{FRET}}(\lambda_{\text{ex,FRET}}, \lambda_{\text{em,FRET}}, FRET) + CFP_{\text{FRET}}(\lambda_{\text{ex,FRET}}, \lambda_{\text{em,FRET}}, direct) \cdot \Gamma} \quad \text{(S1.9)}$$

Here, the factor Γ corresponds to a microscope specific calibration coefficient that accounts for the differences in the emission properties of the donor and acceptor fluorophores including their quantum yields,

$$\Gamma = \frac{F_{\text{FRET}}(A, \lambda_{\text{em,FRET}})}{F_{\text{FRET}}(D, \lambda_{\text{em,FRET}})} \quad \text{(S1.10)}$$

For our experimental setup, Γ is approximately 5.8 assuming ECFP and EYFP FRET pairs. This manipulation effectively converts the sensitized emission component to a corresponding quenching of ECFP fluorescence. The procedure for experimentally deducing Γ factor has been described previously[2,3].

Experimentally, E-FRET efficiency is computed from three cube measurements by substituting Eqs. S1.4 and S1.7 into Eq. S1.9. Thus,

$$E_{\text{D}} = \frac{S_{\text{FRET}}(440,535) - R_{\text{D1}} \cdot S_{\text{CFP}}(440,480) - R_{\text{A1}} \cdot S_{\text{YFP}}(500,530\text{LP})}{S_{\text{FRET}}(440,535) - R_{\text{D1}} \cdot S_{\text{CFP}}(440,480) - R_{\text{A1}} \cdot S_{\text{YFP}}(500,530\text{LP}) + R_{\text{D1}} \cdot S_{\text{CFP}}(440,480) \cdot \Gamma} \quad \text{(S1.11)}$$

In subsequent sections, we consider E_A and E_D for 1:1 donor-acceptor complex and then generalize the relation for a $n_D : n_A$ multimeric complex.

1.2 3^3-FRET and E-FRET efficiencies for 1:1 complexes.

In this section, we demonstrate that for a 1:1 donor-acceptor interaction the maximal 3^3-FRET efficiency ($E_{A,max}$) and E-FRET efficiency ($E_{D,max}$) must equal the true efficiency of energy transfer. We consider the transition diagram shown in **Supplementary Fig. 2** that specifies the fluorescence output of donor and acceptor molecules according to three "subsystems[1]": (1) Excitation subsystem that models the excitation of donor and acceptor fluorophores, (2) fluorophore rate constant subsystem that describes the probability flux between excited and ground states of both donor (D^* and D respectively) and acceptor (A^* and A respectively) molecules including FRET, and (3) emission-detection subsystem that describes the efficacy of detecting the excited donor and acceptor relaxations.

To compute 3^3-FRET efficiency for a 1:1 complex, we first consider the term, $YFP_x(\lambda_{ex,x}, \lambda_{em,x}, \text{direct})$ (yellow branch of **Supplementary Fig. 2b**). In this scenario, as the EYFP molecules are directly excited, only the excitation and subsequent relaxation of the acceptor need to considered. As such, the probability the acceptor molecule is excited through a general optical cube 'x' follows the relation,

$$\frac{dP_{\text{A*}}}{dt} = I_0 \cdot G_{\text{x}}(\text{A}, \lambda_{\text{ex,x}}) \cdot P_{\text{A}} - (k_{\text{A}} + k_{\text{A,nr}}) \cdot P_{\text{A*}} \quad \text{(S1.12)}$$

Here, I_0 is the average intensity of the excitation source. The system is assumed to be in the "low-excitation" limit ($P_A \sim 1$), where the power of the excitation light source is sufficiently low that the steady-state probability of the acceptor being in the ground state is approximately unity.

$$\Rightarrow P_{A*} = \frac{I_0 \cdot G_x(A, \lambda_{ex,x})}{k_A + k_{A,nr}} \tag{S1.13}$$

Next, we consider the efficiency of detecting acceptor fluorophore relaxations through the optical cube 'x'. The rate of acceptor relaxations from a single fluorophore that give rise to fluorescence emission is given by, $k_A \cdot P_{A*}$. The fluorescence output detected by the photomultiplier tube (PMT mV per second) is thus given by,

$$N_A \cdot k_A \cdot P_{A*} \cdot \hat{F}_x(A, \lambda_{em,x}) \tag{S1.14}$$

Here, N_A refers to total number of acceptor molecules and $\hat{F}_x(A, \lambda_{em,x})$ is the output transfer function that converts acceptor relaxations to the PMT output in mV as defined above in Section 1.1.2 above. Substituting Eq. S1.13 into S1.14 and recognizing that $F_x(A, \lambda_{em,x}) = \hat{F}_x(A, \lambda_{em,x}) \cdot QY_A = \hat{F}_x(A, \lambda_{em,x}) \cdot k_A/(k_A + k_{A,nr})$ yields,

$$\begin{aligned} YFP_x(\lambda_{ex,x}, \lambda_{em,x}, direct) &= N_A \cdot k_A \cdot \frac{I_0 \cdot G_x(A, \lambda_{ex,x})}{k_A + k_{A,nr}} \cdot \hat{F}_x(A, \lambda_{em,x}) \\ &= N_A \cdot I_0 \cdot G_x(A, \lambda_{ex,x}) \cdot F_x(A, \lambda_{em,x}) \end{aligned} \tag{S1.15}$$

Next, we consider the term $YFP_x(\lambda_{ex,x}, \lambda_{em,x}, \text{FRET})$, the EYFP emission as a result of FRET from ECFP as measured through optical cube 'x'. To do so, consider the excitation of ECFP molecules that are bound to EYFP (green branch of **Supplementary Fig. 2b**),

$$\frac{dP_{D*}}{dt} = I_0 \cdot G_x(D, \lambda_{ex,x}) \cdot P_D - (k_D + k_{D,nr} + k_T) \cdot P_{D*} \tag{S1.16}$$

Again at steady-state, assuming "low-excitation" limit,

$$P_{D*} = \frac{I_0 \cdot G_x(D, \lambda_{ex,x})}{k_D + k_{D,nr} + k_T} \tag{S1.17}$$

Next, consider the excitation and relaxation of the bound acceptor molecule, the rate of excitation, as a result of FRET from donor, is given by, $k_T \cdot P_{D*}$. Analogous to Eqs. S1.12-S1.13 at steady-state then,

$$P_{A*} = \frac{k_T \cdot P_{D*}}{k_A + k_{A,nr}} = I_0 \cdot G_x(D, \lambda_{ex,x}) \cdot \frac{k_T}{k_D + k_{D,nr} + k_T} \cdot \frac{1}{k_A + k_{A,nr}} \tag{S1.18}$$

The total fluorescence output $YFP_x(\lambda_{ex,x}, \lambda_{em,x}, \text{FRET})$ from EYFP excited by FRET from donor is given by,

$$\begin{aligned} YFP_x(\lambda_{ex,x}, \lambda_{em,x}, \text{FRET}) &= N_B \cdot k_A \cdot I_0 \cdot G_x(D, \lambda_{ex,x}) \cdot \frac{k_T}{k_D + k_{D,nr} + k_T} \cdot \frac{1}{k_A + k_{A,nr}} \cdot \hat{F}_x(A, \lambda_{em,x}) \\ &= N_B \cdot I_0 \cdot G_x(D, \lambda_{ex,x}) \cdot F_x(A, \lambda_{em,x}) \cdot \frac{k_T}{k_D + k_{D,nr} + k_T} \end{aligned} \tag{S1.19}$$

Here, N_B denotes the total number of bound complexes equal to the number of bound donors (N_{Db}) and bound acceptors (N_{Ab}). Recalling that the efficiency of FRET is given by $E = k_T / (k_D + k_{D,nr} + k_T)$, Eq. S1.19 reduces to

$$YFP_x(\lambda_{ex,x}, \lambda_{em,x}, \text{FRET}) = N_B \cdot I_0 \cdot G_x(D, \lambda_{ex,x}) \cdot F_x(A, \lambda_{em,x}) \cdot E \tag{S1.20}$$

Substituting Eqs. S1.15 and S1.20 into Eq. S1.1 yields,

$$E_A = \frac{N_B \cdot I_0 \cdot G_{FRET}(D, \lambda_{ex,FRET}) \cdot F_{FRET}(A, \lambda_{em,FRET}) \cdot E}{N_A \cdot I_0 \cdot G_{FRET}(A, \lambda_{ex,FRET}) \cdot F_{FRET}(A, \lambda_{em,FRET})} \cdot \frac{G_{FRET}(A, \lambda_{ex,FRET})}{G_{FRET}(D, \lambda_{ex,FRET})} \tag{S1.21}$$

$$\Rightarrow E_{\rm A} = \frac{N_{\rm B}}{N_{\rm A}} \cdot E = \frac{N_{\rm Ab}}{N_{\rm A}} \cdot E \qquad \text{(S1.22)}$$

where $N_{\rm B}$ is the number of bound complexes and $N_{\rm Ab}$ is the number of A molecules that are bound ($N_{\rm Ab} = N_{\rm B}$ for 1:1 stoichiometry). The maximal 3^3-FRET, $E_{\rm A,max}$ is attained when all acceptor molecules are bound,

$$E_{\rm A,max} = E \qquad \text{(S1.23)}$$

Thus in the case of 1:1 binding, the maximal 3^3-FRET efficiency ($E_{\rm A,max}$) reports the true efficiency of energy transfer as summarized in main text **Fig. 1a**.

To determine E-FRET efficiency ($E_{\rm D}$), we need to further compute the ECFP intensity due to direct excitation, $CFP_{\rm x}(\lambda_{\rm ex,x}, \lambda_{\rm em,x}, \text{direct})$ (blue and green branches of **Supplementary Fig. 1b**). This term is the sum of fluorescence output from ECFP molecules that are free and ECFP molecules that are bound to an EYFP molecule. First, consider the fraction of ECFP molecules bound to an acceptor, the steady-state probability of such donor molecules being excited is given by Eq. S1.17. The fluorescence output from $N_{\rm B}$ such molecules is,

$$\begin{aligned} N_{\rm B} \cdot k_{\rm D} \cdot P_{\rm D*} \cdot \hat{F}_{\rm x}({\rm D}, \lambda_{\rm em,x}) &= N_{\rm B} \cdot I_0 \cdot G_{\rm x}({\rm D}, \lambda_{\rm ex,x}) \cdot \frac{k_{\rm D}}{k_{\rm D} + k_{\rm D,nr} + k_{\rm T}} \cdot \hat{F}_{\rm x}({\rm D}, \lambda_{\rm em,x}) \\ &= N_{\rm B} \cdot I_0 \cdot G_{\rm x}({\rm D}, \lambda_{\rm ex,x}) \cdot \frac{k_{\rm D} + k_{\rm D,nr}}{k_{\rm D} + k_{\rm D,nr} + k_{\rm T}} \cdot \frac{k_{\rm D}}{k_{\rm D} + k_{\rm D,nr}} \cdot \hat{F}_{\rm x}({\rm D}, \lambda_{\rm em,x}) \\ &= N_{\rm B} \cdot I_0 \cdot G_{\rm x}({\rm D}, \lambda_{\rm ex,x}) \cdot (1 - E) \cdot F_{\rm x}({\rm D}, \lambda_{\rm em,x}) \end{aligned} \qquad \text{(S1.24)}$$

Next, consider ECFP molecules that are free, the probability of excitation of such donor molecules can be determined by a relation akin to Eq. S1.16. Since, these molecules are not bound to EYFP, they cannot undergo FRET. Thus, the probability that such molecules are excited at steady-state in "low-excitation limit" ($P_{\rm D} \sim 1$) is,

$$P_{\rm D*} = \frac{I_0 \cdot G_{\rm x}({\rm D}, \lambda_{\rm ex,x})}{k_{\rm D} + k_{\rm D,nr}} \qquad \text{(S1.25)}$$

The fluorescence output from $N_{\rm Dfree}$ such molecules with rate of fluorescence relaxations $k_{\rm D} \cdot P_{\rm D*}$ is given by,

$$N_{\rm Dfree} \cdot I_0 \cdot G_{\rm x}(D, \lambda_{\rm ex,x}) \cdot \frac{k_{\rm D}}{k_{\rm D} + k_{\rm D,nr}} \cdot \hat{F}_{\rm x}({\rm D}, \lambda_{\rm em,x}) = N_{\rm Dfree} \cdot I_0 \cdot G_{\rm x}(D, \lambda_{\rm ex,x}) \cdot F_{\rm x}({\rm D}, \lambda_{\rm em,x}) \qquad \text{(S1.26)}$$

Thus, the total ECFP intensity due to direct excitation is given by the sum of Eqs. S1.24 and S1.26,

$$\begin{aligned} CFP_{\rm x}(\lambda_{\rm ex,x}, \lambda_{\rm em,x}, \text{direct}) &= N_{\rm B} \cdot I_0 \cdot G_{\rm x}({\rm D}, \lambda_{\rm ex,x}) \cdot (1-E) \cdot F_{\rm x}({\rm D}, \lambda_{\rm em,x}) + N_{\rm Dfree} \cdot I_0 \cdot G_{\rm x}(D, \lambda_{\rm ex,x}) \cdot F_{\rm x}({\rm D}, \lambda_{\rm em,x}) \\ &= N_{\rm D} \cdot I_0 \cdot G_{\rm x}(D, \lambda_{\rm ex,x}) \cdot F_{\rm x}({\rm D}, \lambda_{\rm em,x}) - N_{\rm B} \cdot E \cdot I_0 \cdot G_{\rm x}(D, \lambda_{\rm ex,x}) \cdot F_{\rm x}({\rm D}, \lambda_{\rm em,x}) \end{aligned} \qquad \text{(S1.27)}$$

Note that $N_{\rm D} = N_{\rm Dfree} + N_{\rm B}$. The E-FRET efficiency can be computed by substituting Eqs. S1.27 and Eq. S1.20 into Eq. S1.9. Notice that the denominator, $YFP_{\rm FRET}(\lambda_{\rm ex,FRET}, \lambda_{\rm em,FRET}, \text{FRET}) + \Gamma \cdot CFP_{\rm FRET}(\lambda_{\rm ex,\ FRET}, \lambda_{\rm em,\ FRET}, \text{direct})$ can be simplified to, $N_{\rm D} \cdot I_0 \cdot G_{\rm FRET}({\rm D}, \lambda_{\rm em,FRET}) \cdot F_{\rm FRET}({\rm A}, \lambda_{\rm em,FRET})$. Thus,

$$E_{\rm D} = \frac{N_B \cdot E \cdot I_0 \cdot G_{\rm FRET}(D, \lambda_{\rm ex,FRET}) \cdot F_{\rm FRET}({\rm A}, \lambda_{\rm em,FRET})}{N_D \cdot I_0 \cdot G_{\rm FRET}(D, \lambda_{\rm ex,FRET}) \cdot F_{\rm FRET}({\rm A}, \lambda_{\rm em,FRET})} = \frac{N_B}{N_D} \cdot E \qquad \text{(S1.28)}$$

The maximal E-FRET efficiency occurs when all donor molecules are bound, thus in this limit $N_{\rm B} = N_{\rm D}$,

$$E_{\rm D,max} = E \qquad \text{(S1.29)}$$

Thus, for 1:1 binding, the maximal E-FRET efficiency ($E_{\rm D,max}$) also reports the true efficiency of energy transfer as summarized in main text **Fig. 1a**. Moreover, $E_{\rm D,max} = E = E_{\rm A,max}$ and the stoichiometry ratio (ν) is given by,

$$\upsilon = \frac{E_{\rm A,max}}{E_{\rm D,max}} = 1 \qquad \text{(S1.30)}$$

1.3 3^3-FRET and E-FRET efficiencies for n_D: n_A complexes.

We next consider 3^3-FRET and E-FRET efficiencies for binding interactions with n_D:n_A donor-acceptor stoichiometry. To do so, we consider the modified transition diagram shown in **Supplementary Fig. 3** that models fluorescence output from the system of donors and acceptors are specified according to the three subsystems: excitation, fluorophore-rate-constant, and emission-detection subsystems. Notice that the modified diagram incorporates potential FRET between each pair of n_D and n_A complexes – with $k_{Ti,j}$ representing rate of FRET between i^{th} donor and j^{th} acceptor. Procedurally, we again follow our derivation of 3^3-FRET efficiency and E-FRET efficiency for the 1:1 complex (Section 1.2).

To deduce 3^3-FRET efficiency, we first consider the direct excitation of EYFP molecules – the term $YFP_x(\lambda_{ex,x}, \lambda_{em,x}, \text{direct})$ (yellow branch of **Supplementary Fig. 3b**). Here, since EYFP molecules are directly excited, the probability that the acceptor is excited follows the identical relation determined for 1:1 complexes shown in Eq. S1.13. Accordingly, the total steady-state fluorescence output detected by the PMT is given by Eq. S1.15 assuming "low-excitation limit",

$$YFP_x(\lambda_{ex,x}, \lambda_{em,x}, direct) = N_A \cdot I_0 \cdot G_x(A, \lambda_{ex,x}) \cdot F_x(A, \lambda_{em,x}) \qquad (S1.31)$$

Next, we consider the total EYFP fluorescence output due to FRET from bound ECFP molecules – the term $YFP_x(\lambda_{ex,x}, \lambda_{em,x}, \text{FRET})$ (green branch of **Supplementary Fig. 3b**). Accordingly, consider the excitation of any given donor molecule D_i in the bound complex. The rate of excitation of each donor is $I_0 \cdot G_x(A,\lambda_{ex,x})$. In addition, the donor could relax from the excited state by releasing a photon or through FRET to any of the bound acceptor molecule A_j. Thus,

$$\frac{dP_{D_i*}}{dt} = I_0 \cdot G_x(D, \lambda_{ex,x}) \cdot P_{D_i} - (k_D + k_{D,nr} + k_{Ti,1} + ... + k_{Ti,j} + k_{Ti,nA}) \cdot P_{D_i*} \qquad (S1.32)$$

At steady-state, the probability of the donor being excited assuming "low-excitation" limit ($P_{Di} \sim 1$) is given by,

$$P_{D_i*} = \frac{I_0 \cdot G_x(D, \lambda_{ex,x})}{k_D + k_{D,nr} + \sum_{j=1}^{n_A} k_{Ti,j}} \qquad (S1.33)$$

Next we consider the probability of excitation of jth acceptor as a result of FRET. Since any of the associated donor molecules could transfer energy,

$$\frac{dP_{Aj*}}{dt} = (k_{T1,j} \cdot P_{D1} + ... + k_{Ti,j} \cdot P_{Di} + ... + k_{T1,nD} \cdot P_{D_{nD}}) \cdot P_{Aj} - (k_A + k_{A,nr}) \cdot P_{Aj*} \qquad (S1.34)$$

Assuming steady-state,

$$P_{A_j*} = \frac{\sum_{i=1}^{n_D} k_{Ti,j} \cdot P_{D_i}}{k_A + k_{A,nr}} \qquad (S1.35)$$

Substituting Eq. S1.33 into Eq. S1.35 yields,

$$P_{A_j*} = \frac{1}{k_A + k_{A,nr}} \cdot \sum_{i=1}^{n_D} k_{Ti,j} \cdot \frac{I_0 \cdot G_x(D, \lambda_{ex,x})}{k_D + k_{D,nr} + \sum_{j=1}^{n_A} k_{Ti,j}} = \frac{I_0 \cdot G_x(D, \lambda_{ex,x})}{k_A + k_{A,nr}} \cdot \sum_{i=1}^{n_D} E_{i,j} \qquad (S1.36)$$

Importantly, since any of the n_A acceptor molecules in a bound complex could emit photons, the total YFP output due to FRET would be an aggregate of all such relaxations. Thus,

$$YFP_x(\lambda_{ex,x}, \lambda_{em,x}, \text{FRET}) = N_B \cdot \sum_{j=1}^{n_A} k_A \cdot P_{A_j*} \cdot \hat{F}_x(A, \lambda_{em,x}) = N_B \cdot k_A \cdot \hat{F}_x(A, \lambda_{em,x}) \cdot \sum_{j=1}^{n_A} P_{A_j*} \qquad (S1.37)$$

Substituting Eq. S1.36 into Eq. S1.37 and recalling that $F_x(\mathrm{A}, \lambda_{em,x}) = \hat{F}_x(\mathrm{A}, \lambda_{em,x}) \cdot QY_A = \hat{F}_x(\mathrm{A}, \lambda_{em,x}) \cdot k_A/(k_A + k_{A,nr})$ yields the relation,

$$YFP_x(\lambda_{ex,x}, \lambda_{em,x}, \mathrm{FRET}) = N_B \cdot I_0 \cdot G_x(\mathrm{D}, \lambda_{ex,x}) \cdot F_x(\mathrm{A}, \lambda_{em,x}) \cdot \sum_{j=1}^{n_A} \sum_{i=1}^{n_D} E_{i,j} \quad \text{(S1.38)}$$

Reassuringly, in the case of a single donor and acceptor, Eq. S1.38 reduces to the equation shown in Eq. S1.20.

The 3^3-FRET efficiency can then be deduced by substituting Eqs. S1.31 and S1.38 into Eq. S1.1 assuming measurements are obtained using the FRET cube. Thus,

$$E_A = \frac{N_B \cdot I_0 \cdot G_x(\mathrm{D}, \lambda_{ex,x}) \cdot F_x(\mathrm{A}, \lambda_{em,x}) \cdot \sum_{j=1}^{n_A} \sum_{i=1}^{n_D} E_{i,j}}{N_A \cdot I_0 \cdot G_{FRET}(\mathrm{A}, \lambda_{ex,FRET}) \cdot F_{FRET}(\mathrm{A}, \lambda_{em,FRET})} \cdot \frac{G_{FRET}(A, \lambda_{ex,FRET})}{G_{FRET}(D, \lambda_{ex,FRET})}$$

$$= \frac{N_B}{N_A} \cdot \sum_{j=1}^{n_A} \sum_{i=1}^{n_D} E_{i,j} \quad \text{(S1.39)}$$

The maximal 3^3-FRET efficiency is attained when all acceptor molecules are bound (i.e. $N_{Afree} \sim 0$). Since $N_A = N_{Afree} + n_A \cdot N_B$, when all acceptors are bound, $N_B = N_A / n_A$. Thus, the maximal 3^3-FRET efficiency is given by,

$$E_{A,max} = \frac{1}{n_A} \cdot \sum_{j=1}^{n_A} \sum_{i=1}^{n_D} E_{i,j} \quad \text{(S1.40)}$$

Thus, in the most general case of n_D:n_A binding interaction, the maximal 3^3-FRET efficiency reports the expected number of energy transfer events per acceptor molecule given the donor molecules are accepted. Reassuringly again, if the binding is 1:1, then Eq. S1.40 reduces to the true FRET efficiency between the donor and acceptor molecules.

To determine E-FRET efficiency, we next compute $CFP_x(\lambda_{ex,x}, \lambda_{em,x}, \text{direct})$, the ECFP intensity due to direct excitation. Since both free and acceptor-bound donor molecules can release photons, the total CFP intensity is the sum of these two pathways. For the bound complex, the each donor molecule within the complex could potentially emit a photon with rate of relaxations being $k_D \cdot P_{Di*}$. Thus, the net fluorescence output from donor molecules within the bound complex is the sum of fluorescence emission from each constituent donor,

$$N_B \cdot \sum_{i=1}^{n_D} k_D \cdot P_{D_i*} \cdot \hat{F}_x(\mathrm{D}, \lambda_{em,x}) \quad \text{(S1.41)}$$

Substituting the probability that a given donor fluorophore in the bound complex is excited (Eq. S1.33) yields,

$$N_B \cdot \sum_{i=1}^{n_D} k_D \cdot \frac{I_0 \cdot G_x(\mathrm{D}, \lambda_{ex,x})}{k_D + k_{D,nr} + \sum_{j=1}^{n_A} k_{Ti,j}} \cdot \hat{F}_x(\mathrm{D}, \lambda_{em,x}) \quad \text{(S1.42)}$$

$$= N_B \cdot I_0 \cdot G_x(\mathrm{D}, \lambda_{ex,x}) \cdot \sum_{i=1}^{n_D} \frac{k_D + k_{D,nr}}{k_D + k_{D,nr} + \sum_{j=1}^{n_A} k_{Ti,j}} \cdot \frac{k_D}{k_D + k_{D,nr}} \hat{F}_x(\mathrm{D}, \lambda_{em,x})$$

Simplifying this relation further one obtains,

$$N_B \cdot I_0 \cdot G_x(\mathrm{D}, \lambda_{ex,x}) \cdot F_x(\mathrm{D}, \lambda_{em,x}) \cdot \sum_{i=1}^{n_D} \left(1 - \frac{\sum_{j=1}^{n_A} k_{Ti,j}}{k_D + k_{D,nr} + \sum_{j=1}^{n_A} k_{Ti,j}} \right) \quad \text{(S1.43)}$$

Recognizing that for each donor D_i, $E_{i,j} = k_{Ti,j} / (k_D + k_{D,nr} + k_{Ti,1} + \ldots + k_{Ti,j} + \ldots + k_{Ti,A})$ Eq. S1.43 simplifies to,

$$N_B \cdot I_0 \cdot G_x(D, \lambda_{ex,x}) \cdot F_x(D, \lambda_{em,x}) \cdot \sum_{i=1}^{n_D} \left(1 - \sum_{j=1}^{n_A} E_{i,j}\right)$$
$$= N_B \cdot I_0 \cdot G_x(D, \lambda_{ex,x}) \cdot F_x(D, \lambda_{em,x}) \cdot \left(n_D - \sum_{i=1}^{n_D}\sum_{j=1}^{n_A} E_{i,j}\right) \quad \text{(S1.44)}$$

Next, the fluorescence output from N_{Dfree} free donor molecules that do not undergo FRET is given by Eq. S1.26. Thus, the total CFP fluorescence from bound and unbound donor molecules is given by,

$$CFP_x(\lambda_{ex,x}, \lambda_{em,x}, \text{direct}) = N_B \cdot I_0 \cdot G_x(D, \lambda_{ex,x}) \cdot F_x(D, \lambda_{em,x}) \cdot \left(n_D - \sum_{i=1}^{n_D}\sum_{j=1}^{n_A} E_{i,j}\right)$$
$$+ N_{Dfree} \cdot I_0 \cdot G_x(D, \lambda_{ex,x}) \cdot F_x(D, \lambda_{em,x}) \quad \text{(S1.45)}$$

Since the total number of donor molecules, $N_D = n_D \cdot N_B + N_{Dfree}$, Eq. S1.45 reduces to,

$$CFP_x(\lambda_{ex,x}, \lambda_{em,x}, \text{direct}) = N_D \cdot I_0 \cdot G_x(D, \lambda_{ex,x}) \cdot F_x(D, \lambda_{em,x}) - N_B \cdot I_0 \cdot G_x(D, \lambda_{ex,x}) \cdot F_x(D, \lambda_{em,x}) \sum_{i=1}^{n_D}\sum_{j=1}^{n_A} E_{i,j} \quad \text{(S1.46)}$$

The E-FRET efficiency could be computed by substituting Eq. S1.46 and Eq. S1.38 into Eq. S1.9 assuming measurements are obtained using the FRET cube. Accordingly,

$$E_D = \frac{N_B \cdot I_0 \cdot G_x(D, \lambda_{ex,x}) \cdot F_x(A, \lambda_{em,x}) \cdot \sum_{j=1}^{n_A}\sum_{i=1}^{n_D} E_{i,j}}{N_D \cdot I_0 \cdot G_{FRET}(D, \lambda_{ex,FRET}) \cdot F_{FRET}(A, \lambda_{em,FRET})} = \frac{N_B}{N_D} \cdot \sum_{j=1}^{n_A}\sum_{i=1}^{n_D} E_{i,j} \quad \text{(S1.47)}$$

The maximal E-FRET efficiency ($E_{D,max}$) is attained when all donor molecules are bound. Under this condition, $N_D = n_D \cdot N_B$ and the maximal FRET efficiency is then given by,

$$E_{D,max} = \frac{1}{n_D} \cdot \sum_{j=1}^{n_A}\sum_{i=1}^{n_D} E_{i,j} \quad \text{(S1.48)}$$

Of note, these results can also be derived from the alternate definition of E_D (Eq. S1.8). To do so, note that $CFP_{FRET|before} = CFP_x(\lambda_{ex,x}, \lambda_{em,x}, \text{direct})$.

$$CFP_{FRET|after} = N_D \cdot I_0 \cdot G_x(D, \lambda_{ex,x}) \cdot F_x(D, \lambda_{em,x}) \quad \text{(S1.49)}$$

This expression is akin to Eq. S1.31 for acceptors and can be determined in a like fashion by considering CFP output without any possible FRET. Substituting Eq. S1.46 and Eq. S1.49 will also yield Eq. S1.48.

The stoichiometry ratio for $n_D : n_A$ binding interaction is given by the ratio of Eq. S1.40 and Eq. S1.48,

$$\upsilon = \frac{E_{A,max}}{E_{D,max}} = \frac{\frac{1}{n_A} \cdot \sum_{j=1}^{n_A}\sum_{i=1}^{n_D} E_{i,j}}{\frac{1}{n_D} \cdot \sum_{j=1}^{n_A}\sum_{i=1}^{n_D} E_{i,j}} = \frac{n_D}{n_A} \quad \text{(S1.50)}$$

SUPPLEMENTARY NOTE 2

Extended statistical analysis to evaluate stoichiometry of CaM binding to holochannel complexes.

In this section, we furnish statistical analysis to evaluate the stoichiometry of CaM binding to $Ca_V1.2$ and $Na_V1.4$ channels based on measurements of maximal 3^3-FRET and E-FRET efficiencies. Accordingly, we utilize two sample t-test to systematically evaluate the null hypothesis that the observed $E_{A,max}$ and $E_{D,max}$ values are consistent with a given stoichiometry. Thus, for a proposed stoichiometry $r = n_D / n_A$ = 1/3, 1/2, 1, 2, 3, etc the null and alternate hypothesis are as follows:

$$H_0: \quad E_{A,max} - r \cdot E_{A,max} = 0$$
$$H_0: \quad E_{A,max} - r \cdot E_{A,max} \neq 0$$

We further establish a stringent criterion of $p < 0.01$ to reject the null hypothesis. Note that it is possible that with this analysis, multiple null hypotheses may be accepted. In such a case, we may conclude that we have insufficient statistical power with our measurements to establish a single stoichiometry.

For binding of CaM to the $Ca_V1.2$ channel, **Supplementary Fig. 8a** shows the probability that the experimentally observed $E_{A,max}$ and $E_{D,max}$ correspond to an interaction with a given stoichiometry $r = n_D / n_A$ under low Ca^{2+} (black curve) or high Ca^{2+} (red curve) conditions. The green shaded area denotes the region of significance where the null hypothesis is accepted. As evident, under low Ca^{2+} conditions, the observed maximal FRET efficiencies are most consistent with 1:1 stoichometry, while alternative hypothesis such as 1/3, 1/2, 2, and 3 can all be rejected with high degree of confidence. The *p*-values are reported in table below.

For binding of CaM to the $Na_V1.4$ channels, our data are most consistent with a 1:1 stoichiometry of interaction under both basal and elevated Ca^{2+} conditions (**Supplementary Fig. 8b**). In particular, despite the low absolute magnitude of the maximal FRET efficiencies, the *p*-values computed show that the likelihood for the interaction to be of 1:1 stoichiometry is ~500× (low Ca^{2+}) and ~ 1000× (high Ca^{2+}) more likely than a 2:1 CaM:channel interaction. These results strongly suggest the absence of Ca^{2+}-dependent shift in CaM/channel interaction stoichiometry for Na channels.

	$r = n_D / n_A$				
Binding Pair	1/3	1/2	1	2	3
$Ca_V1.2$ / apoCaM	reject (p = 2.2E-4)	reject (p = 1.6E-4)	accept (p = 0.082)	reject (p = 3.2E-5)	reject (p = 1.6E-6)
$Ca_V1.2$ / Ca^{2+}/CaM	reject (p = 2.0E-4)	reject (p = 3.0E-4)	reject (p = 3.7E-3)	accept (p = 0.7)	reject (p = 9.7E-4)
$Na_V1.4$ / apoCaM	reject (p = 2.2E-4)	reject (p = 5.5E-3)	accept (p = 0.081)	reject (p = 1.9E-4)	reject (p = 8.7E-6)
$Na_V1.4$ / Ca^{2+}/CaM	reject (p = 7.2E-4)	reject (p = 1.9E-3)	accept (p = 0.34)	reject (p = 3.4E-4)	reject (p = 1.8E-5)

SUPPLEMENTARY NOTE 3

Effect of maturation of fluorescent proteins on apparent FRET efficiency measurements.

A confounding factor in determining maximal FRET efficiencies is incomplete or slow maturation of fluorescent proteins. While both EYFP and ECFP are thought to mature relatively quickly and independently with high efficacy, it is possible that the presence of a small fraction of immature fluorophores could diminish the measured apparent FRET efficiencies. Here, we evaluate $E_{A,max}$ and $E_{D,max}$ as a function of the fraction of mature of donors ($f_{m,D}$) and acceptors ($f_{m,A}$) in a cell.

Effect of immature fluorophores on $E_{A,max}$ and $E_{D,max}$ of 1:1 complexes. For 3^3-FRET efficiencies, consider the YFP signal as a result of direct excitation through the YFP cube (Eq. S1.31). Since only the mature fraction of acceptors ($f_{m.A}$) can emit photons,

$$YFP_x(\lambda_{ex,x}, \lambda_{em,x}, direct) = f_{m,A} \cdot N_A \cdot I_0 \cdot G_x(A, \lambda_{ex,x}) \cdot F_x(A, \lambda_{em,x}) \quad (S3.1)$$

N_A is the number of acceptors. $G_x(A, \lambda_{ex,x})$ and $F_x(A, \lambda_{ex,x})$ are calibration constants define in Section 1.1.2. To determine YFP emission due to FRET excitation, we note that FRET occurs if and only if both the donor and the acceptor are mature. The steady-state probability for an excited acceptor is then,

$$P_{A*} = I_0 \cdot G_x(D, \lambda_{ex,x}) \cdot \frac{k_T}{k_D + k_{D,nr} + k_T} \cdot \frac{1}{k_A + k_{A,nr}} \cdot f_{m,D} \cdot f_{m,A} \quad (S3.2)$$

The product $f_{m.A} \cdot f_{m.A}$ is the probability that both the donor and the acceptor in the complex are mature. Given that FRET efficiency, $E = k_T / (k_D + k_{D,nr} + k_T)$, the YFP signal output due to FRET from all bound complexes (S1.19) is modified to be,

$$YFP_x(\lambda_{ex,x}, \lambda_{em,x}, \text{FRET}) = N_B \cdot I_0 \cdot G_x(D, \lambda_{ex,x}) \cdot F_x(A, \lambda_{em,x}) \cdot E \cdot f_{m,D} \cdot f_{m,A} \quad (S3.3)$$

The 3^3-FRET efficiency is the ratio of $YFP_x(\lambda_{ex}, \lambda_{em,x}, \text{FRET}) / YFP_x(\lambda_{ex}, \lambda_{em,x}, \text{direct})$ (for $f_{m,A} > 0$),

$$E_A = f_{m,D} \cdot E \cdot N_B / N_A = f_{m,D} \cdot A_b \cdot E \quad (S3.4)$$

Of note, the E_A is only affected by maturation of the donor fluorophore and not that of the acceptor as noted previously[4]. The maximal 3^3-FRET efficiency is attained when all acceptors are bound ($N_B = N_A$; $A_b = 1$),

$$E_{A,max} = f_{m,D} \cdot E \quad (S3.5)$$

An analogous argument can be made for E-FRET efficiency[5]. The steady-state probability that an unbound donor is excited is given by,

$$P_{D*} = \frac{I_0 \cdot G_x(D, \lambda_{ex,x})}{k_D + k_{D,nr}} \cdot f_{m,D} \quad (S3.6)$$

The fluorescence output for N_{Dfree} molecules is,

$$N_{Dfree} \cdot f_{m,D} \cdot I_0 \cdot G_x(D, \lambda_{ex,x}) \cdot \frac{k_D}{k_D + k_{D,nr}} \cdot \hat{F}_x(D, \lambda_{em,x}) \quad (S3.7)$$
$$= N_{Dfree} \cdot f_{m,D} \cdot I_0 \cdot G_x(D, \lambda_{ex,x}) \cdot F_x(D, \lambda_{em,x})$$

Next, consider the bound fraction of donors – the probability that the donor in the complex is excited is,

$$P_{D*} = \frac{I_0 \cdot G_x(D, \lambda_{ex,x})}{k_D + k_{D,nr} + k_T} \cdot f_{m,D} \cdot f_{m,A} + \frac{I_0 \cdot G_x(D, \lambda_{ex,x})}{k_D + k_{D,nr}} \cdot f_{m,D} \cdot (1 - f_{m,A}) \quad (S3.8)$$

The fluorescence output for N_B such molecules is given by,

$$N_{B} \cdot k_{D} \cdot P_{D*} \cdot \hat{F}_{x}(D, \lambda_{em,x}) = N_{B} \cdot I_{0} \cdot G_{x}(D, \lambda_{ex,x}) \cdot \hat{F}_{x}(D, \lambda_{em,x}) \cdot \left(\frac{k_{D}}{k_{D} + k_{D,nr} + k_{T}} \cdot f_{m,D} \cdot f_{m,A} + \frac{k_{D}}{k_{D} + k_{D,nr}} \cdot f_{m,D} \cdot (1 - f_{m,A}) \right)$$

$$= N_{B} \cdot I_{0} \cdot G_{x}(D, \lambda_{ex,x}) \cdot F_{x}(D, \lambda_{em,x}) \cdot \left((1-E) \cdot f_{m,D} \cdot f_{m,A} + f_{m,D} \cdot (1 - f_{m,A}) \right)$$

$$= N_{B} \cdot I_{0} \cdot G_{x}(D, \lambda_{ex,x}) \cdot F_{x}(D, \lambda_{em,x}) \cdot f_{m,D} \cdot \left(1 - E \cdot f_{m,A} \right) \qquad \text{(S3.9)}$$

Combining Eqs. S3.7 and S3.9, the total CFP output is,

$$CFP_{x}(\lambda_{ex,x}, \lambda_{em,x}, \text{direct}) = N_{D} \cdot f_{m,D} \cdot I_{0} \cdot G_{x}(D, \lambda_{ex,x}) \cdot F_{x}(D, \lambda_{em,x}) - N_{B} \cdot f_{m,D} \cdot f_{m,A} \cdot E \cdot I_{0} \cdot G_{x}(D, \lambda_{ex,x}) \cdot F_{x}(D, \lambda_{em,x}) \qquad \text{(S3.10)}$$

Notice that $YFP_{FRET}(\lambda_{ex,FRET}, \lambda_{em,FRET}, \text{FRET}) + \Gamma \cdot CFP_{FRET}(\lambda_{ex,\,FRET}, \lambda_{em,\,FRET})$ simplifies to

$$N_{D} \cdot f_{m,D} \cdot I_{0} \cdot G_{x}(D, \lambda_{ex,x}) \cdot F_{x}(D, \lambda_{em,x}) \qquad \text{(S3.11)}$$

The E-FRET efficiency is the ratio of Eq. S3.3 and Eq. S3.11,

$$E_{D} = E \cdot f_{m,A} \cdot N_{B} / N_{D} = f_{m,A} \cdot D_{b} \cdot E \qquad \text{(S3.12)}$$

Interestingly, the E-FRET efficiency is insensitive to the fraction of mature donors but instead depends upon the fraction of the mature acceptors. The maximal E-FRET efficiency ($E_{D,max}$) is attained when all donor species are bound (Note, some donors may be bound to an immature acceptor).

$$E_{D,max} = f_{m,A} \cdot E \qquad \text{(S3.13)}$$

The stoichiometry ratio, ν, is the ratio of Eqs. S1.53 and S1.61,

$$\upsilon = \frac{E_{A,max}}{E_{D,max}} = \frac{f_{m,D}}{f_{m,A}} \cdot 1 = \rho \cdot 1 \qquad \text{(S3.14)}$$

This result argues that for 1:1 interactions, the error in measured stoichiometry due to immature fluorophores depends on the relative proportion of mature donors to acceptors ($\rho = f_{m,D} / f_{m,A}$) in cells. This error can be experimentally determined through analysis of stoichiometry ratio for various CFP-YFP dimers (main text Fig. 2). In addition, we evaluated stoichiometry ratio for one additional dimeric construct where CFP and YFP bookend CaM ($E_{A,max} / E_{D,max} = 0.957$). On average, we find $\rho = f_{m,D} / f_{m,A} = 1.026$ implying that the bias in stoichiometry estimates due to immature CFP and YFP would be < 3%, a negligible amount.

We undertook stochastic simulations to validate this theoretical analysis. We simulate 40000 molecular complexes in a single cell containing exactly 1 donor and 1 acceptor with each donor or acceptor is assigned to be mature or immature at random with a certain probability (donor, $f_{m,D}$ and acceptor, $f_{m,A}$). The YFP signal due to FRET (Eq. S1.19), the YFP signal due to direct excitation (Eq. S1.15), and the CFP signal (Eq. S1.27) from all 40000 molecules are estimated. From these three quantities, the apparent 3^3-FRET and E-FRET efficiencies are computed using Eq. S1.1 and Eq. S1.9. If either a donor or an acceptor is immature, then $k_T = 0$. If both the

donor and the acceptor are mature then a random value of k_T is assumed (k_T / (k_D + $k_{Dn,r}$) < 0.5) for a given simulation. Supplementary Fig. 9a-c furnishes results from one among twenty simulations. Fitting with Eq. S3.5, for 1:1 complexes, our simulations show that $E_{A,max}$ is linearly dependent on the fraction of mature donors ($f_{m,D}$) in a cell (Supplementary Fig. 9a). That said, $E_{D,max}$ is linearly dependent on the fraction of mature acceptors ($f_{m,A}$) in a cell (see Eq. S3.13; Supplementary Fig. 9b). Importantly, the simulations show that the stoichiometry ratio, $\nu = E_{A,max} / E_{D,max}$, can reliably estimate interaction stoichiometry if both donor and acceptor fluorophores have greater than 90% folding efficiency (Supplementary Fig. 9c, red shaded area). For lower folding efficiencies, our assay can still assess interaction stoichiometry so long as both the donor and the acceptor have similar maturation efficiencies, i.e. $\rho = f_{m,D} / f_{m,A} \sim 1$ (Supplementary Fig. 9c; red contour, $\nu = 1$). The experimental value of $\rho = f_{m,D} / f_{m,A} = 1.026$ for CFP–YFP pair is plotted as a gray dotted line for comparison. Notice that this line closely follows the red contour ($\nu = 1$) where the ratio of FRET efficiencies corresponds to the actual interaction stoichiometry.

Effect of immature fluorophores on $E_{A,max}$ and $E_{D,max}$ of n_D:1 complexes. We next evaluate the effect of immature fluorophores on multimeric complexes involving multiple donors and a single acceptor as is the case with experiments in main text Fig. 3 and 4b.

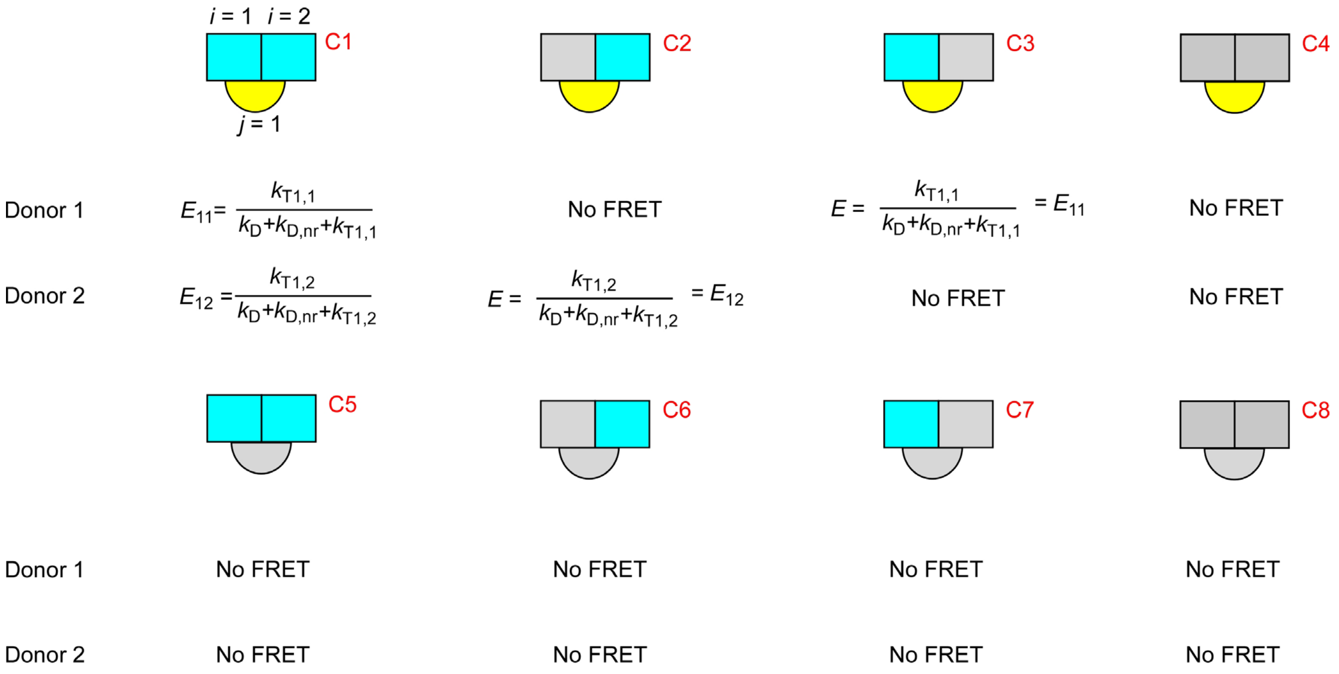

Effect of immature fluorophores on pairwise FRET efficiencies. Various bound species containing multiple donors and a single acceptor with incomplete fluorophore maturation. All fluorophores are mature in the C1 complex. The pairwise FRET efficiency between donor 1 and the acceptor is E_{11}, while that between donor 2 and the acceptor is E_{21}. For complexes C2 and C3, exactly one donor is mature. While the immature donor cannot participate in FRET, the FRET efficiency between the mature donors and the acceptor in the complex is unaffected by the presence of an immature acceptor. If the acceptor is immature or if both donors are immature, then FRET is not possible.

First, the total YFP output due to direct excitation is given by,

$$YFP_x(\text{direct}, \lambda_{ex,x}, \lambda_{em,x}) = N_A \cdot f_{m,A} \cdot I_0 \cdot G_x(A, \lambda_{ex,x}) \cdot F_x(A, \lambda_{em,x}) \tag{S3.15}$$

Next, we consider the YFP signal due to FRET assuming that any of the fluorophores in the complex may be immature. Importantly, for an n_D:1 complex, there are 2^{n_D+1} possible classes of molecular complexes where a

donor or the acceptor may be immature. An example for a 2:1 interaction is enumerated in the figure above. The total fluorescence output is the superposition of fluorescence signal from each class of such complexes and could be considered individually as follows.

If the acceptor in the complex is not mature, there is no FRET. Similarly for complexes with a mature acceptor, if the associated donors are immature, no FRET is again possible. However, if exactly one donor in the complex is mature, this donor could participate in FRET with the mature acceptor. The total YFP output due to FRET for such complexes is given by,

$$YFP_x(FRET,\lambda_{ex,x},\lambda_{em,x};m_D = 1, m_A = 1)$$
$$= N_\mathrm{B}\cdot I_0\cdot G_\mathrm{x}(D,\lambda_\mathrm{ex,x})\cdot F_\mathrm{x}(\mathrm{A},\lambda_\mathrm{em,x})\cdot\left(f_\mathrm{m,D}^1(1-f_\mathrm{m,D})^{n_\mathrm{D}-1}\right)\cdot\left(f_\mathrm{m,A}\right)\cdot\sum_{i=1}^{n_\mathrm{D}}E_\mathrm{i,1} \tag{S3.16}$$

Here, $f_\mathrm{m,D}^1(1-f_\mathrm{m,D})^{n_\mathrm{D}-1}$ is the probability that the donor at position i is mature while all other donors in the complex immature and $f_\mathrm{m,A}$ is the probability that the acceptor is mature. Note that this derivation parallels Eq. S3.3, but incorporates the possibility of multiple donors. For complexes with exactly 2 mature donors and a mature acceptor,

$$YFP_x(FRET,\lambda_{ex,x},\lambda_{em,x};m_D = 2, m_A = 1)$$
$$= N_\mathrm{B}\cdot I_0\cdot G_\mathrm{x}(D,\lambda_\mathrm{ex,x})\cdot F_\mathrm{x}(\mathrm{A},\lambda_\mathrm{em,x})\cdot\left(\binom{n_\mathrm{D}-1}{1}f_\mathrm{m,D}^2(1-f_\mathrm{m,D})^{n_\mathrm{D}-2}\right)\cdot\left(f_\mathrm{m,A}\right)\cdot\sum_{i=1}^{n_\mathrm{D}}E_\mathrm{i,1} \tag{S3.17}$$

Here, $\binom{n_\mathrm{D}-1}{1}f_\mathrm{m,D}^2(1-f_\mathrm{m,D})^{n_\mathrm{D}-2}$ is the probability that two donors namely the ith donor and exactly one other donor is mature in the bound complex. This equation can be generalized as,

$$YFP_x(FRET,\lambda_{ex,x},\lambda_{em,x};m_D = \varphi, m_A = 1)$$
$$= N_\mathrm{B}\cdot I_0\cdot G_\mathrm{x}(D,\lambda_\mathrm{ex,x})\cdot F_\mathrm{x}(\mathrm{A},\lambda_\mathrm{em,x})\cdot\left(\binom{n_\mathrm{D}-1}{\varphi-1}f_\mathrm{m,D}^\varphi(1-f_\mathrm{m,D})^{n_\mathrm{D}-\varphi}\right)\cdot\left(f_\mathrm{m,A}\right)\cdot\sum_{i=1}^{n_\mathrm{D}}E_\mathrm{i,1} \tag{S3.18}$$

Finally, if all donors were mature,

$$YFP_x(FRET,\lambda_{ex,x},\lambda_{em,x};m_D = n_\mathrm{D}, m_A = 1)$$
$$= N_\mathrm{B}\cdot I_0\cdot G_\mathrm{x}(D,\lambda_\mathrm{ex,x})\cdot F_\mathrm{x}(\mathrm{A},\lambda_\mathrm{em,x})\cdot\left(f_\mathrm{m,D}^{n_\mathrm{D}}\right)\cdot\left(f_\mathrm{m,A}\right)\cdot\sum_{i=1}^{n_\mathrm{D}}E_\mathrm{i,1} \tag{S3.19}$$

The total YFP output from all complexes is given by,

$$YFP_x(FRET,\lambda_{ex,x},\lambda_{em,x}) = \sum_{\varphi=0}^{n_\mathrm{D}}YFP_x(FRET,\lambda_{ex,x},\lambda_{em,x};m_D = \varphi, m_A = 1) \tag{S3.20}$$

$$= N_{\mathrm{B}} \cdot I_0 \cdot G_{\mathrm{x}}(D,\lambda_{\mathrm{ex,x}}) \cdot F_{\mathrm{x}}(\mathrm{A},\lambda_{\mathrm{em,x}}) \cdot \sum_{\varphi=1}^{n_{\mathrm{D}}} \binom{n_{\mathrm{D}}-1}{\varphi-1} f_{\mathrm{m,D}}^{\varphi} \cdot (1-f_{\mathrm{m,D}})^{n_{\mathrm{D}}-\varphi} \cdot \left(f_{\mathrm{m,A}}\right) \cdot \sum_{i=1}^{n_{\mathrm{D}}} E_{\mathrm{i},1}$$

$$= N_{\mathrm{B}} \cdot I_0 \cdot G_{\mathrm{x}}(D,\lambda_{\mathrm{ex,x}}) \cdot F_{\mathrm{x}}(\mathrm{A},\lambda_{\mathrm{em,x}}) \cdot \left(f_{\mathrm{m,D}} \cdot f_{\mathrm{m,A}} \cdot \sum_{i=1}^{n_{\mathrm{D}}} E_{\mathrm{i},1} \right) \cdot \underbrace{\left(\sum_{\varphi=1}^{n_{\mathrm{D}}} \binom{n_{\mathrm{D}}-1}{\varphi-1} f_{\mathrm{m,D}}^{\varphi-1} \cdot (1-f_{\mathrm{m,D}})^{n_{\mathrm{D}}-\varphi} \right)}_{=1 \text{ by binomial expansion of } (1-f_{\mathrm{m,D}}+f_{\mathrm{m,D}})^{n_{\mathrm{D}}-1}}$$

This expression simplifies to,

$$YFP_x(FRET,\lambda_{ex,x},\lambda_{em,x}) = N_{\mathrm{B}} \cdot I_0 \cdot G_{\mathrm{x}}(D,\lambda_{\mathrm{ex,x}}) \cdot F_{\mathrm{x}}(\mathrm{A},\lambda_{\mathrm{em,x}}) \cdot f_{\mathrm{m,D}} \cdot f_{\mathrm{m,A}} \cdot \sum_{i=1}^{n_{\mathrm{D}}} E_{\mathrm{i},1} \quad \text{(S3.21)}$$

Next, we estimate the CFP fluorescence output due to direct excitation. The total CFP output is the superposition of CFP signal from different classes of complexes where a variable number of fluorophores are immature. First consider CFP molecules that are unbound to an acceptor ($N_{\mathrm{Dfree}} = N_{\mathrm{D}} - n_{\mathrm{D}} \cdot N_{\mathrm{B}}$). The probability that the free donor fluorophore is mature is $f_{\mathrm{m,D}}$. The total fluorescence output from free CFP is given by,

$$CFP_x(direct,\lambda_{ex,x},\lambda_{em,x};m_A=0) \;=\; N_{\mathrm{Dfree}} \cdot f_{\mathrm{m,D}} \cdot I_0 \cdot G_{\mathrm{x}}(D,\lambda_{\mathrm{ex,x}}) \cdot F_{\mathrm{x}}(\mathrm{D},\lambda_{\mathrm{em,x}}) \quad \text{(S3.22)}$$

Consider complexes where the acceptor is immature but may contain 1 to n_{D} mature donors. The expected number of mature CFP molecules in complexes with an immature acceptor is $N_{\mathrm{B}} \cdot (1-f_{\mathrm{m,A}}) \cdot f_{\mathrm{m,D}} \cdot n_{\mathrm{D}}$. The CFP fluorescence output will be,

$$CFP_x(direct,\lambda_{ex,x},\lambda_{em,x};m_A=0) = N_{\mathrm{B}} \cdot (1-f_{\mathrm{m,A}}) \cdot n_{\mathrm{D}} \cdot f_{\mathrm{m,D}} \cdot I_0 \cdot G_{\mathrm{x}}(D,\lambda_{\mathrm{ex,x}}) \cdot F_{\mathrm{x}}(\mathrm{D},\lambda_{\mathrm{em,x}}) \quad \text{(S3.23)}$$

Next we turn to complexes containing a mature acceptor. If there is exactly one mature donor, then this donor can undergo FRET with the acceptor. The CFP fluorescence output for such complexes is (similar to Eq. S1.44),

$$\begin{aligned} &CFP_x(direct,\lambda_{ex,x},\lambda_{em,x};m_{\mathrm{D}}=1,m_A=1) \\ &= N_{\mathrm{B}} \cdot (f_{\mathrm{m,D}} \cdot (1-f_{\mathrm{m,D}})^{n_{\mathrm{D}}-1}) \cdot f_{\mathrm{m,A}} \cdot I_0 \cdot G_{\mathrm{x}}(D,\lambda_{\mathrm{ex,x}}) \cdot F_{\mathrm{x}}(\mathrm{D},\lambda_{\mathrm{em,x}}) \cdot \sum_{i=1}^{n_{\mathrm{D}}} (1-E_{\mathrm{i},1}) \\ &= N_{\mathrm{B}} \cdot (f_{\mathrm{m,D}} \cdot (1-f_{\mathrm{m,D}})^{n_{\mathrm{D}}-1}) \cdot f_{\mathrm{m,A}} \cdot I_0 \cdot G_{\mathrm{x}}(D,\lambda_{\mathrm{ex,x}}) \cdot F_{\mathrm{x}}(\mathrm{D},\lambda_{\mathrm{em,x}}) \cdot n_{\mathrm{D}} \\ &\quad - N_{\mathrm{B}} \cdot (f_{\mathrm{m,D}} \cdot (1-f_{\mathrm{m,D}})^{n_{\mathrm{D}}-1}) \cdot f_{\mathrm{m,A}} \cdot I_0 \cdot G_{\mathrm{x}}(D,\lambda_{\mathrm{ex,x}}) \cdot F_{\mathrm{x}}(\mathrm{D},\lambda_{\mathrm{em,x}}) \sum_{i=1}^{n_{\mathrm{D}}} E_{\mathrm{i},1} \end{aligned} \quad \text{(S3.24)}$$

This formula can be generalized for complexes containing φ mature donors,

$$\begin{aligned} &CFP_x(direct,\lambda_{ex,x},\lambda_{em,x};m_{\mathrm{D}}=\varphi,m_A=1) \\ &= N_{\mathrm{B}} \cdot \left(\binom{n_{\mathrm{D}}\text{-}1}{\varphi-1} \cdot f_{\mathrm{m,D}}^{\varphi} \cdot (1-f_{\mathrm{m,D}})^{n_{\mathrm{D}}-\varphi} \right) \cdot f_{\mathrm{m,A}} \cdot I_0 \cdot G_{\mathrm{x}}(D,\lambda_{\mathrm{ex,x}}) \cdot F_{\mathrm{x}}(\mathrm{D},\lambda_{\mathrm{em,x}}) \cdot n_{\mathrm{D}} \\ &\quad - N_{\mathrm{B}} \cdot \left(\binom{n_{\mathrm{D}}\text{-}1}{\varphi-1} \cdot f_{\mathrm{m,D}}^{\varphi} \cdot (1-f_{\mathrm{m,D}})^{n_{\mathrm{D}}-\varphi} \right) \cdot f_{\mathrm{m,A}} \cdot I_0 \cdot G_{\mathrm{x}}(D,\lambda_{\mathrm{ex,x}}) \cdot F_{\mathrm{x}}(\mathrm{D},\lambda_{\mathrm{em,x}}) \cdot \sum_{i=1}^{n_{\mathrm{D}}} E_{\mathrm{i},1} \end{aligned} \quad \text{(S3.25)}$$

The total CFP fluorescence output for bound complexes containing a mature acceptor is given by,

$$CFP_x(direct,\lambda_{ex,x},\lambda_{em,x};m_A=1) = \sum_{\varphi=1}^{n_{\mathrm{D}}} CFP_x(direct,\lambda_{ex,x},\lambda_{em,x};m_A=\varphi,m_A=1) \quad \text{(S3.26)}$$

Substituting Eq. S3.25 into Eq. S3.26,

$$CFP_x(direct, \lambda_{ex,x}, \lambda_{em,x}; m_A = \varphi, m_A = 1)$$

$$= \sum_{\varphi=1}^{n_D} N_B \cdot \left(\binom{n_D - 1}{\varphi - 1} \cdot f_{m,D}^{\varphi} \cdot (1 - f_{m,D})^{n_D - \varphi} \right) \cdot f_{m,A} \cdot I_0 \cdot G_x(D, \lambda_{ex,x}) \cdot F_x(D, \lambda_{em,x}) \cdot n_D$$

$$- \sum_{\varphi=1}^{n_D} N_B \cdot \left(\binom{n_D - 1}{\varphi - 1} \cdot f_{m,D}^{\varphi} \cdot (1 - f_{m,D})^{n_D - \varphi} \right) \cdot f_{m,A} \cdot I_0 \cdot G_x(D, \lambda_{ex,x}) \cdot F_x(D, \lambda_{em,x}) \cdot \sum_{i=1}^{n_D} E_{i,1} \quad \text{(S3.27)}$$

This expression can be simplified using binomial theorem (similar to Eq. S3.20),

$$CFP_x(direct, \lambda_{ex,x}, \lambda_{em,x}; m_A = 1) = N_B \cdot f_{m,A} \cdot f_{m,D} \cdot I_0 \cdot G_x(D, \lambda_{ex,x}) \cdot F_x(D, \lambda_{em,x}) \cdot n_D$$

$$- N_B \cdot f_{m,A} \cdot f_{m,D} \cdot I_0 \cdot G_x(D, \lambda_{ex,x}) \cdot F_x(D, \lambda_{em,x}) \sum_{i=1}^{n_D} E_{i,1} \quad \text{(S3.28)}$$

Finally, the total CFP output from the cell is the sum of fluorescence emission from (1) unbound CFP (Eq. S3.22), (2) CFP bound to immature acceptor (Eq. S3.23), and (3) CFP bound to a mature acceptor (Eq. S3.28).

$$CFP_x(direct, \lambda_{ex,x}, \lambda_{em,x}) = N_D \cdot f_{m,D} \cdot I_0 \cdot G_x(D, \lambda_{ex,x}) \cdot F_x(D, \lambda_{em,x})$$

$$- N_B \cdot f_{m,A} \cdot f_{m,D} \cdot I_0 \cdot G_x(D, \lambda_{ex,x}) \cdot F_x(D, \lambda_{em,x}) \sum_{i=1}^{n_D} E_{i,1} \quad \text{(S3.29)}$$

Note, $N_D = N_{Dfree} + n_D \cdot N_B$. The 3^3-FRET efficiency (E_A) can be computed based on its definition (Eq. S1.1) by computing the ratio of YFP emissions from FRET (Eq. S3.21) compared to direct excitation (Eq. S3.15).

$$E_A = \frac{N_B}{N_A} \cdot f_{m,D} \cdot \sum_{i=1}^{n_D} E_{i,1} \quad \text{(S3.30)}$$

The maximal 3^3-FRET efficiency is attained when all acceptors are bound, i.e. $N_A = n_A \cdot N_B = N_B$.

$$E_{A,max} = f_{m,D} \cdot \sum_{i=1}^{n_D} E_{i,1} \quad \text{(S3.31)}$$

Similarly E-FRET efficiency can be computed from its definition by substituting Eq. S3.15 and Eq. S3.29 into Eq. S1.9. Notice, $CFP_x(direct, \lambda_{ex,x}, \lambda_{em,x}) + \Gamma \cdot YFP_x(FRET, \lambda_{ex,x}, \lambda_{em,x}) = N_D \cdot f_{m,D} \cdot I_0 \cdot G_x(D, \lambda_{ex,x}) \cdot F_x(D, \lambda_{em,x})$

$$E_D = \frac{N_B}{N_A} \cdot f_{m,A} \cdot \sum_{i=1}^{n_D} E_{i,1} \quad \text{(S3.32)}$$

The maximal E-FRET efficiency is attained when all donors are bound; thus, $N_D = n_D \cdot N_B$.

$$E_{D,max} = f_{m,A} \cdot \frac{1}{n_D} \cdot \sum_{i=1}^{n_D} E_{i,1} \quad \text{(S3.33)}$$

The stoichiometry ratio can be deduced by dividing Eq. S3.31 by S3.33,

$$\upsilon = \frac{E_{A,max}}{E_{D,max}} = \frac{f_{m,D}}{f_{m,A}} \cdot \frac{n_D}{n_A} = \rho \cdot \frac{n_D}{1} \quad \text{(S3.34)}$$

This result parallels our finding for 1:1 interactions. The FRET-based stoichiometry ratio misestimates the actual interaction stoichiometry simply as the ratio of maturation efficiency for the donor versus the acceptor, ρ. Stochastic simulations of complexes with 3:1 and 6:1 complexes are shown in Supplementary Fig. 9d-f and

Supplementary Fig. 9g-i where the rate of energy transfers were chosen to match experimental data shown for binding of CFP-tagged CaM to YFP-tagged Myosin Va tandem IQ domains (main text Fig. 3). Once again, we found that $E_{A,max}$ was linearly dependent on the fraction of mature donors though this measure was largely insensitive to the fraction of mature acceptors. By contrast, $E_{D,max}$ was linearly dependent on fraction of mature acceptors while insensitive to the fraction of mature donors. Our analysis shows that if both donors and acceptors had >90% folding efficiency, the assay could without ambiguity discern 3:1 and 6:1 binding interactions. Further simulations showed that with 90% efficiency, the stoichiometry ratio could reliably distinguish up to 8:1 complexes. This range could be extended further if the folding efficiencies of donor and acceptor are similar ($\rho \sim 1$) as is true for the CFP / YFP FRET pair ($\rho \sim 1.026$).

This analysis can be generalized to complexes of n_D:n_A stoichiometry. For such complexes, the measured stoichiometry ratio (ν) is the product of the ratio of fractional maturation of donors and acceptors and the interaction stoichiometry,

$$\upsilon = \frac{E_{A,max}}{E_{D,max}} = \frac{f_{m,D}}{f_{m,A}} \cdot \frac{n_D}{n_A} = \rho \cdot \frac{n_D}{n_A} \qquad \text{(S3.35)}$$

To validate this result, we undertook stochastic simulations using MATLAB to characterize the effect of immature donors and acceptors on complexes containing multiple acceptors. To this end, we analyzed $E_{A,max}$ and $E_{D,max}$ for complexes of n_D:n_A = 1:3 (Supplementary Fig. 9j-l) and 1:6 (Supplementary Fig. 9m-o) stoichiometries as a function of $f_{m,A}$ and $f_{m,D}$. For both complexes, $E_{A,max}$ is linearly dependent on the fraction of mature donors ($f_{m,D}$; black line, Supplementary Fig. 9j,m) while largely insensitive to the fraction of mature acceptors ($f_{m,A}$; black line, Supplementary Fig. 9k, n) particularly when $f_{m,A}$ > 50%. By contrast, $E_{D,max}$ is sensitive to the fraction of mature acceptors (red trace, Supplementary Fig. 9k, n). Importantly, if both donors and acceptors have greater than 90% folding efficiency, then the ratio ν robustly estimates the actual interaction stoichiometry.

Supplementary References

1 Erickson, M. G., Alseikhan, B. A., Peterson, B. Z. & Yue, D. T. Preassociation of calmodulin with voltage-gated Ca(2+) channels revealed by FRET in single living cells. *Neuron* **31**, 973-985 (2001).

2 Chen, H., Puhl, H. L., 3rd, Koushik, S. V., Vogel, S. S. & Ikeda, S. R. Measurement of FRET efficiency and ratio of donor to acceptor concentration in living cells. *Biophysical journal* **91**, L39-41, doi:10.1529/biophysj.106.088773 (2006).

3 Ben-Johny, M., Yang, P. S., Bazzazi, H. & Yue, D. T. Dynamic switching of calmodulin interactions underlies Ca2+ regulation of CaV1.3 channels. *Nature communications* **4**, 1717, doi:10.1038/ncomms2727 (2013).

4 Erickson, M. G., Moon, D. L. & Yue, D. T. DsRed as a potential FRET partner with CFP and GFP. *Biophysical journal* **85**, 599-611, doi:10.1016/S0006-3495(03)74504-4 (2003).

5 Wlodarczyk, J. *et al.* Analysis of FRET signals in the presence of free donors and acceptors. *Biophysical journal* **94**, 986-1000, doi:10.1529/biophysj.107.111773 (2008).
